# Complete sequence and comparative genomic analysis of eight native *Pseudomonas syringae* plasmids belonging to the pPT23A family

**DOI:** 10.1186/s12864-017-3763-x

**Published:** 2017-05-10

**Authors:** José A. Gutiérrez-Barranquero, Francisco M. Cazorla, Antonio de Vicente, George W. Sundin

**Affiliations:** 1 Instituto de Hortofruticultura Subtropical y Mediterránea La Mayora (IHSM-UMA-CSIC), Departamento de Microbiología, Facultad de Ciencias, Universidad de Málaga, Málaga, Spain; 2 Department of Plant, Soil, and Microbial Sciences, Michigan State University, East Lansing, MI 48824 USA

**Keywords:** Copper resistance, *rulAB*, *hopBD1*, Plasmid phylogeny, *P. syringae*, Genomic island

## Abstract

**Background:**

The pPT23A family of plasmids appears to be indigenous to the plant pathogen *Pseudomonas syringae* and these plasmids are widely distributed and widely transferred among pathovars of *P. syringae* and related species. pPT23A-family plasmids (PFPs) are sources of accessory genes for their hosts that can include genes important for virulence and epiphytic colonization of plant leaf surfaces. The occurrence of repeated sequences including duplicated insertion sequences on PFPs has made obtaining closed plasmid genome sequences difficult. Therefore, our objective was to obtain complete genome sequences from PFPs from divergent *P. syringae* pathovars and also from strains of *P. syringae* pv. syringae isolated from different hosts.

**Results:**

The eight plasmids sequenced ranged in length from 61.6 to 73.8 kb and encoded from 65 to 83 annotated orfs. Virulence genes including type III secretion system effectors were encoded on two plasmids, and one of these, pPt0893-29 from *P. syringae* pv. tabaci, encoded a wide variety of putative virulence determinants. The PFPs from *P. syringae* pv. syringae mostly encoded genes of importance to ecological fitness including the *rulAB* determinant conferring tolerance to ultraviolet radiation. Heavy metal resistance genes encoding resistance to copper and arsenic were also present in a few plasmids. The discovery of part of the chromosomal genomic island GI6 from *P. syringae* pv. syringae B728a in two PFPs from two *P. syringae* pv. syringae hosts is further evidence of past intergenetic transfers between plasmid and chromosomal DNA. Phylogenetic analyses also revealed new subgroups of the pPT23A plasmid family and confirmed that plasmid phylogeny is incongruent with *P. syringae* pathovar or host of isolation. In addition, conserved genes among seven sequenced plasmids within the same phylogenetic group were limited to plasmid-specific functions including maintenance and transfer functions.

**Conclusions:**

Our sequence analysis further revealed that PFPs from *P. syringae* encode suites of accessory genes that are selected at species (universal distribution), pathovar (interpathovar distribution), and population levels (intrapathovar distribution). The conservation of type IV secretion systems encoding conjugation functions also presumably contributes to the distribution of these plasmids within *P. syringae* populations.

**Electronic supplementary material:**

The online version of this article (doi:10.1186/s12864-017-3763-x) contains supplementary material, which is available to authorized users.

## Background

*Pseudomonas syringae* is a widely-studied plant-pathogenic bacterium of interest as a model for analysis of the molecular bases of host-pathogen interactions and of epiphytic growth on plant leaf surfaces. This species can be subdivided into over 60 pathological variants or pathovars that are mainly distinguished by host range [1]. Phylogenetic and taxonomic analyses of the *P. syringae* species have demonstrated that the species is grouped into four clades with some former pathovars such as *P. cannabina* and *P. savastanoi* re-elevated into separate species with their own subsets of pathovars [2, 3]. Genetic and genomic analyses of host range differentiation among *P. syringae* pathovars typically focus on differences in the repertoire of specific type III effector proteins [4, 5]. The type III secretion system (T3SS) is required for *P. syringae* pathogenesis and encodes a specialized delivery system functioning in the translocation of effector proteins directly into the cytoplasm of plant cells. Type III effectors function collectively to suppress plant defenses and to establish infections that result in plant cell death and release of nutrients to invading pathogen cells [6–8]. Genome sequence evidence indicates that many type III effector genes were acquired via horizontal gene transfer; in addition, the location of many of these genes adjacent to mobile genetic elements facilitates their transfer to and relocation within genomes [5, 9–11].

Plasmids are critical sources for evolution due to their capacity to acquire foreign DNA sequences and to their ability to affect their transfer among bacteria via self-encoded mechanisms such as conjugation and mobilization [12]. Plasmids are viewed as major contributors to genome innovation and in many cases represent the first acquisition point for foreign DNA sequences into a cell. Plasmid sequences comprise a significant portion of the variable genome (also known as the accessory or flexible genome), that portion of the genome that does not encode basic survival functions but instead contributes to ecological fitness in specific habitats, lifestyles, or environmental conditions [13–15]. Bacterial plasmids tend to encode more mobile genetic elements than chromosomes, and undergo recombination at higher frequencies than chromosomes [16, 17]. These facets of plasmid biology, along with the dispensability of plasmids to organism survival, empower plasmids as breeding grounds for the evolution of novel genetic determinants and for the distribution of these genes within a communal gene pool [12, 18].

The pPT23A plasmid family comprises a group of plasmids that appear to be indigenous to *P. syringae* and related organisms. pPT23A-family plasmids (PFPs) typically range from 35 to 100 kb and share a conserved major replication gene *repA* and origin of replication [19, 20]. The gene content of PFPs encompasses genes encoding plasmid-specific functions, including two distinct gene sets encoding type IV secretion systems (MPF_T_ and MPF_I_ of the eight different classes recently identified [21, 22]) specifying conjugation functions, and a variety of other genes either experimentally shown to or postulated to confer virulence or ecological fitness traits [15]. Sequence and hybridization analyses have shown that individual PFPs can encode genes that are distributed at population, pathovar, or species-specific levels [21, 23–25]. Horizontal gene transfer has played a significant role in the evolution of PFPs; for example, a phylogenetic analysis demonstrated that PFPs were widely transferred among *P. syringae* pathovar hosts and that individual genes had been transferred among distinct PFPs [26]. PFPs have also acted as a repository for the acquisition of bactericide-resistance genes including copper resistance determinants and the streptomycin-resistance transposon Tn*5393* [27–29].

To date, closed genomic sequences of twelve PFPs are available from *P. syringae* pathovars phaseolicola, maculicola, syringae, tomato and actinidiae, *P. cannabina* pathovar (pv.) alisalensis, and *P. savastanoi* pv. savastanoi [3, 23, 25, 30–35]. However, acquisition of closed plasmid sequences has been difficult due to the presence of multiple repeated insertion sequence elements on some of these plasmids and due to the presence of multiple PFPs in many *P. syringae* strains [25]. Draft sequences of various *P. syringae* genomes are becoming available at a faster and faster rate [5, 35–39]. Unfortunately, the presence of plasmids and especially of PFPs in these genomes and an examination of their gene content is lacking. For example, in a comparative genomics study of *P. syringae* pathovars, it was noted that it was “difficult to truly identify presence of plasmids using short-read assembly information alone” [5]. In fact, we only found one *P. syringae* genomics study with significant information on plasmid genome content; results from this study indicated that most of the strain-specific genes identified in *P. savastanoi* pv. glycinea strains were located within plasmid DNA sequences [36].

We hypothesized that further understanding of the evolution of the pPT23A plasmid family and the role of these plasmids in *P. syringae* biology and pathogenesis required the determination and analysis of additional complete, closed plasmid genome sequences. Thus, in this work we sequenced eight PFPs and performed comprehensive bioinformatic analyses of these plasmids.

## Results

### Complete closed native plasmid sequences analysis

It is well known that the majority of strains belonging to the *P. syringae* group can harbor at least one indigenous plasmid [39, 40], although the number of harbored PFP plasmids can vary [19, 24, 25]. In addition, PFPs have been shown to share a large amount of repeated sequences including insertion sequence elements [41]. These two facts can constitute a relevant bottleneck during plasmid isolation and sequencing [5, 25].

In light of the above mentioned potential problems, we selected eight different *P. syringae* strains for this study that were previously identified as harboring only one plasmid, in order to facilitate plasmid isolation and the subsequent sequencing process. Thus, the complete closed sequences of eight different plasmids isolated from eight different *P. syringae* bacterial strains belonging to three pathovars were obtained (Table 1). Six of the eight plasmids were obtained from strains of the pathovar syringae isolated from diseased mango trees (*n* = 4), sweet cherry (*n* = 1), and ornamental pear (*n* = 1). One plasmid was obtained from the pathovar garcae isolated from coffee, and the remaining plasmid was obtained from the pathovar tabaci isolated from tobacco. All of the plasmids analyzed in this study were previously identified to be PFPs because they share the essential replication protein RepA [15, 19]. After plasmid isolation and purification using CsCl-Ethidium bromide gradients, sequencing was carried out using the Roche 454 GS (FLX procedure) platform with paired-end reads. Pass filter reads (PF reads) ranged from 42,807 of pPg2708 to 119,949 of pPs1029, and the mean read length ranged from 249 of pPs6-9 to 335 of pPs0158. Contigs generated for the majority of plasmids were 1 with the exception of pPg2708 plasmid that were 2. In order to verify if the sequencing and subsequent assembly process were reliable, two different test methods were carried out. Firstly, a PCR analysis using specific primers (Additional file 1: Table S1, Additional file 2: Figure S1) designed on the plasmid initial and final raw sequences revealed that the 454 sequencing platform properly provided the closed circular plasmid sequences. Second, the complete sequence of the plasmid pPs0158 obtained by two different sequencing platforms was compared using the BLAST 2 sequences tool from NCBI (last revision November 2016). This nucleotide comparison showed that the plasmid sequences were identical (data not shown), and thus, validated the sequencing method used in this study.

**Table 1:** *Pseudomonas syringae* strains used in this study

*P. syringae*	Origin	Host	Reference or source
pv. syringae			
UMAF0081	Spain	Mango	28
UMAF0170	Spain	Mango	28
UMAF0158	Spain	Mango	28
UMAF1029	Spain	Mango	28
6–9	USA	Sweet Cherry	89
7B44	USA	Ornamental Pear	43
pv. garcae			
2708	Africa	Coffee	NCPPB^a^
pv. tabaci			
0893–29	Hungary	Tobacco	43

^a^ NCPPB, National Collection of Plant Pathogenic Bacteria (United Kingdom)

**Fig. 1:**
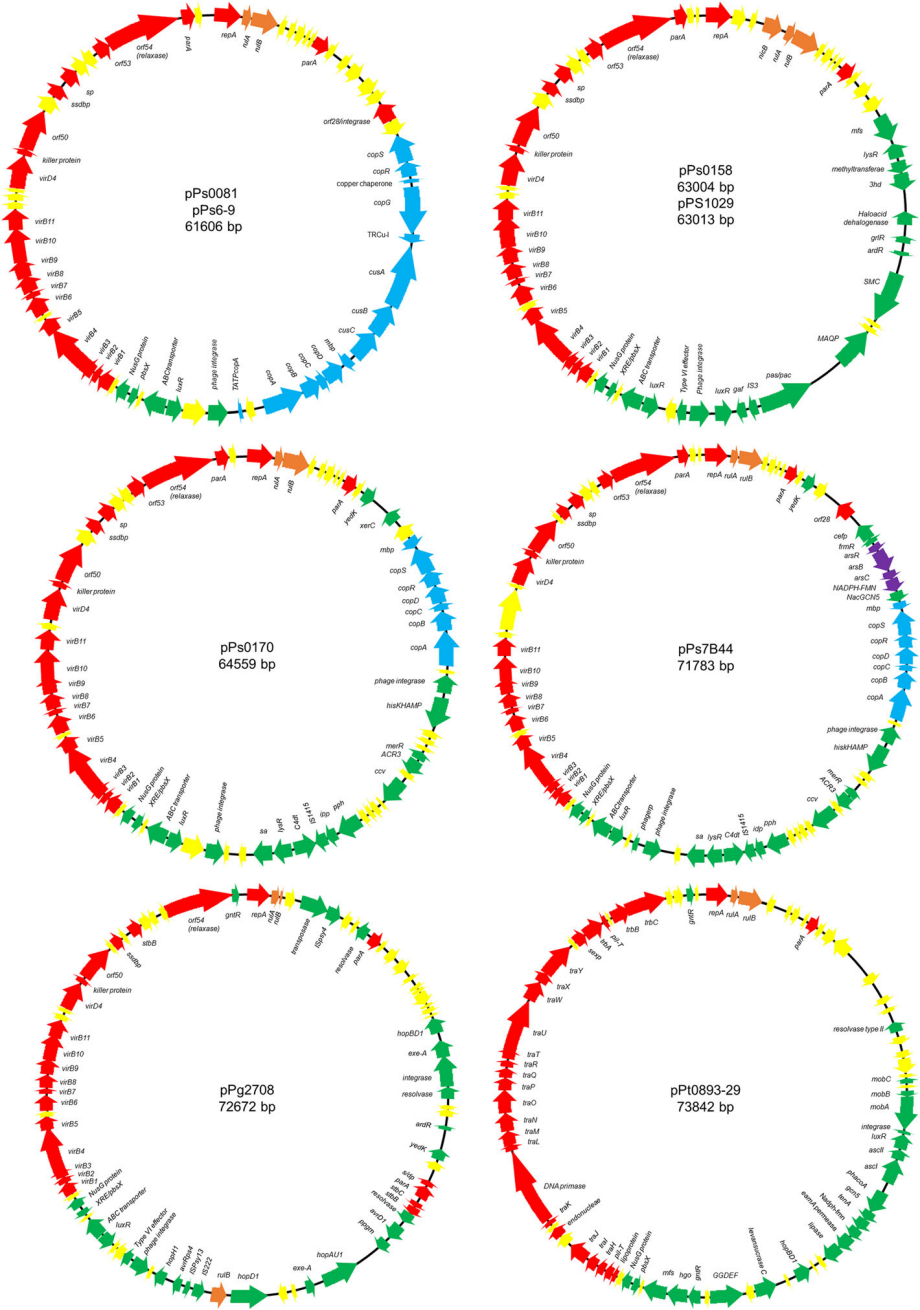
Circular genetic maps of pPs0081, pPs0170, pPs0158, pPs1029, pPs6-9, pPs7B44, pPg2708 and pPt0893-29 plasmids. *repA* is defined as the ORF1 in all plasmids analysis in this study. ORFs are color-coded according to their putative functions. ORFs involved in replication, stability and conjugation functions are colored in *red*. ORFs involved in epiphytic fitness and P. syringae host-interactions are colored in *green*. ORFs involved in copper detoxification are colored in *blue*. ORFs involved in arsenic detoxification are colored in *purple*. The *rulAB* operon is colored in *orange*. ORFs predicted as hypothetical proteins are colored in *yellow*. Putative directions of transcription of each ORF, are indicated by an *arrow*. The identity of each ORF is given inside the plasmid circular map, with the exception of the hypothetical proteins. Plasmids maps are not drawn at scale

The main characteristics of the different plasmids sequenced in this study and their genetic maps are summarized in Table 2 and Fig. 1 respectively. The full length closed circular DNA sequences ranged from 61,606 bp of pPs0081 and pPs6-9 plasmids, to 73,842 bp of the pPt0893-29 plasmid and the %GC content varied from 53.5% of the plasmid pPg2708, to 56.0% of the pPt0893-29 plasmid. Annotation methods revealed that the number of ORFs presented in the different plasmids ranged from 65 to 83, and the percentage of hypothetical proteins present in each plasmid ranged from 32.5 to 37.3%. To assign putative functions to the ORFs predicted in each different plasmid, the sequence of each one was compared with the GenBank database using BLASTx searches showing the homology to previously known gene sequences (Additional file 3: Tables S2-S9). The putative function of the different ORFs and their representations in genetic maps (Fig. 1) indicated the presence of common and different genes between all the plasmids. Using the multiple genome alignment software MAUVE, we determined that the synteny of six (pPs0081, pPs0170, pPs6-9, pPs0158, pPs1029 and pPs7B44) of the eight plasmids was very high (Additional file 4: Figure S2). The pPg2708 plasmid had some differences showing some rearrangement events, although this plasmid did share the type IVA secretion system (MPF_T_, new classification [22]) and some genes related with maintenance and conjugation functions with the other six plasmids analyzed. The highest difference was shown by the pPt0893-29 plasmid; apart from some rearrangement events in comparison with the other plasmids, this plasmid carried the type IVB secretion system (MPF_I_, new classification [22]). Interestingly, the physical co-localization of both different type IV systems was almost identical in all the plasmid sequences. Schematic representation of the type IV MPF_T_ and type IV MPF_I_ secretion systems from the sequenced plasmids, showed that they were practically identical to the type IV MPF_T_ system from the plasmid pPSR1 from *P. syringae* pv. syringae A2 and with the type IV MPF_I_ system from the plasmid DC3000A from *P. syringae* pv. tomato DC3000, respectively (Additional file 5: Figure S3). Small differences could be identified only based on the presence or absence of some hypothetical proteins among the complete set of type IV genes.

**Table 2:** Main characteristics of native plasmids sequenced in this study

*P. syringae* strains	Plasmid name	Plasmid size (bp)	*copABCD*	*rulAB*	Type IVSS	%GC content	n° of ORF’s	Hp (n°-%) ^a^
pv. syringae								
UMAF0081	pPs0081	61,606	+^c^	+^c^	IVA (*virB* genes) ^c^	55.52	66	23–34.8
UMAF0170	pPs0170	64,559	+^c^	+^c^	IVA (*virB* genes)	54.90	75	27–36.0
UMAF0158	pPs0158	63,004	-^c^	+	IVA (*virB* genes)	54.58	65	22–33.8
UMAF1029	pPs1029	63,013	-^c^	+	IVA (*virB* genes)	54.59	65	22–33.8
6–9	pPs6-9	61,606	+^c^	+	IVA (*virB* genes)	55.52	66	23–34.8
7B44	pPs7B44	71,783	+^c^	+	IVA (*virB* genes)	54.97	83	27–32.5
pv. garcae								
2708	pPg2708	72,672	-^c^	+^b^	IVA (*virB* genes)	53.47	83	31–37.3
pv. tabaci								
0893–29	pPt0893-29	73,842	-^c^	+^c^	IVB (*tra* genes)	56.01	81	29–35.8

^a^ Hp, number and percentage of hypothetical protein (number of hypothetical proteins divided by total number of proteins) present in each plasmid

^b^ The *rulA* and *rulB* genes are located in different positions in the plasmid sequence and in different orientations

^c^ Phenotypic analyses performed previously of highlighted plasmids have demonstrated copper resistance (*copABCD*), UV radiation tolerance (*rulAB*), and conjugation transfer (*virB* genes) [28, 29, 43, 88, 45, 89]

Nucleotide comparisons of the eight native plasmids revealed that pPs0081 and pPs6-9 were identical, although both were isolated from different plant hosts and from different continents. In addition, pPs0158 and pPs1029 showed few nucleotide sequence differences, being considered virtually identical plasmids. When the sequences from pPs0170 and pPs7B44 were compared (Additional file 6: Figure S4), both plasmids contained a portion of the genomic island GI6 from *P. syringae* pv. syringae B728a (marked in grey and with dotted green lines), with the difference that pPs7B44 also harbored an operon involved in arsenic resistance (purple arrows), that is present in the GI6 genomic island but missing from pPs0170.

### Comparative analysis of PFP gene content

**Fig. 2:**
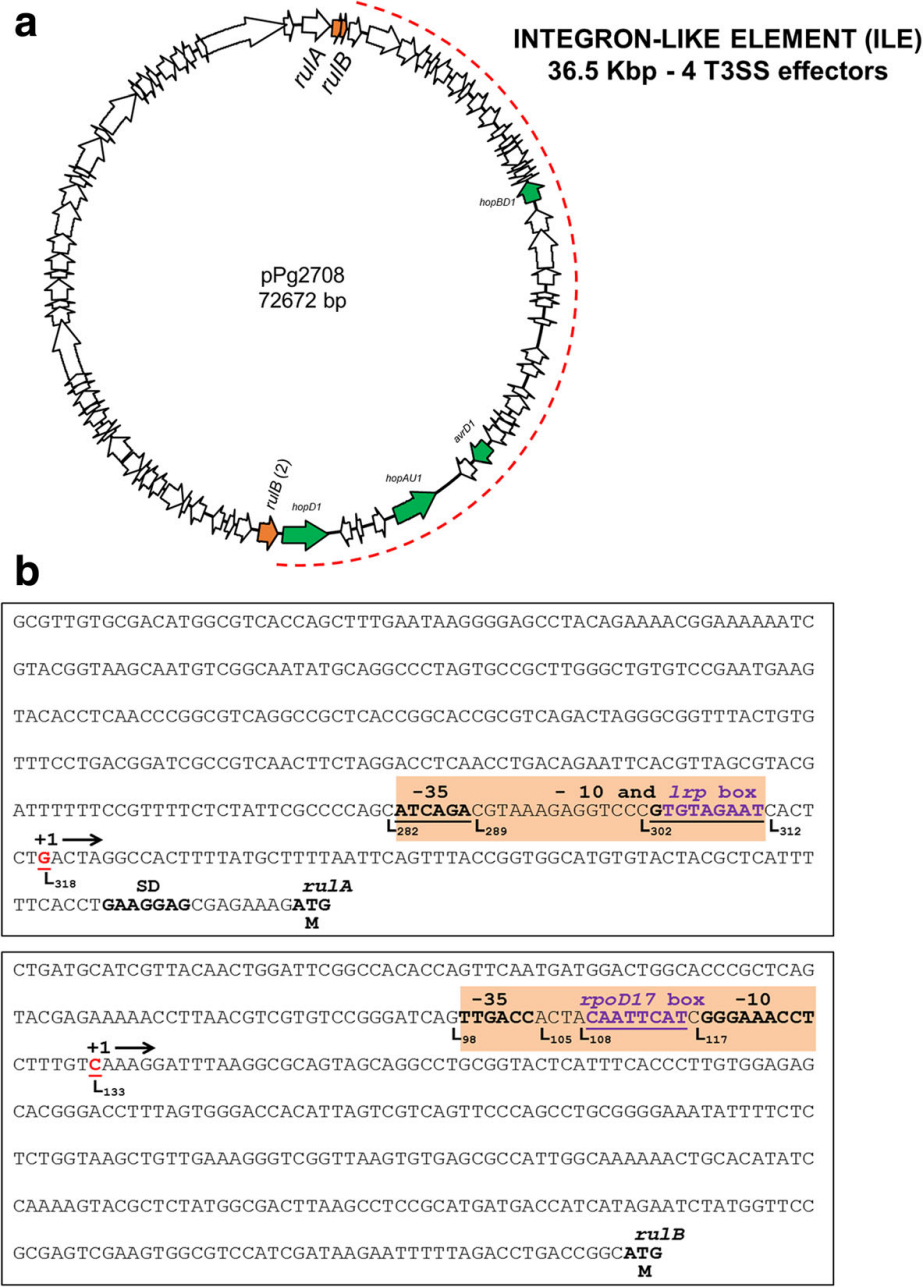
*rulAB* DNA region analysis of pPg2708. **a** Plasmid map of pPg2708 with the location of *rulA* and *rulB* nicked genes, marked in *light orange*. INTEGRON-LIKE ELEMENT (ILE) OF 36.5 Kbp among the *rulB* nicked gene marked with dotted *red line*. Four putative type III effectors are marked in *green color*. **b** Representation of the non-coding region of the *rulA* and the big part of *rulB* genes which contains the putative promoter sequences. The nucleotide sequences of both putative promoters (indicated in *light orange*) showing the proposed −10 box, −35 box and the TF binding sites; the nucleotide position is also indicated. The putative Shine-Dalgarno sequence was only found for the *rulA* gene, and is shown in *bold type*

Many PFPs from *P. syringae* strains have been described previously to improve the epiphytic fitness of their hosts. For example, the *rulAB* operon that functions in DNA repair, confers UV radiation tolerance and contributes to *P. syringae* survival on leaf surfaces [41–44]. The characteristic *rulAB* operon was identified in seven of the eight plasmids sequenced in this study. In the pPg2708 plasmid, the *rulA* gene is present in the same location and orientation compared to the other plasmids, but the *rulB* gene appeared nicked and the two different fragments (one very small, 36 amino acids encoded in the N-terminal region) were located in different positions and in different orientations into the plasmid. Among these two *rulB* fragments, we have found an insertion of DNA of approximately 36.5 Kbp that harbors 4 different effectors of the T3SS (Fig. 2a).

Furthermore, in order to decipher if these genes could be putatively functional, the presence of the promoter and Shine-Dalgarno sequences were bioinfomatically sought for *rulA* and for the largest fragment of *rulB* (Fig. 2b). Promoter regions were determined for both *rulA* and *rulB*, but a Shine-Dalgarno sequence was only found for the *rulA* gene.

Other important features commonly associated with PFPs that improve the epiphytic fitness of *P. syringae* in agricultural ecosystems are the copper resistance genes [28, 45]*.* Although normally copper resistance genes appear forming the *copABCD* operon in *P. syringae*, different genetic structures have also been observed and characterized for their role in improving copper resistance [29]. Two of the eight plasmids sequenced in this study (pPs0170 and pPs7B44) encoded the common *copABCDRS* operon, and a small metal binding protein that is slightly different in sizes between these two plasmids and that has not been previously described associated with this operon in plasmids. Two other plasmids (pPs0081 and pPs6-9) contained a novel genetic structure with the *cusCBA* operon, a *copG* gene, a copper chaperone, and a copper transcriptional regulator inserted into the *copABCDRS* operon; these determinants are related with the detoxification of monovalent cations (Fig. 1).

Plasmid conjugation has been described to be encoded by the type IV secretion system, and is the main mechanism for gene transfer among phytopathogenic bacteria [46]. It was noteworthy that some specific genes not observed previously in other plasmids forming a characteristic structure have been identified in seven of the eight plasmid sequences (not found in pPt0893-29). These genes were located upstream of the type IV MPF_T_ secretion system genes and were delimited by a phage integrase. The arrangement of these genes was the following: 1) a *luxR* transcriptional regulator 2) an *ABC* transporter substrate-binding protein, factor, 3) a *XRE/Pbsx* transcriptional regulator, 4) a NusG transcriptional regulator-termination protein, and then, the type IV MPF_T_ secretion system (Fig. 1).

Finally, PFP sequences have also been related with virulence function, mainly through encoding T3SS effectors and toxin genes [15, 23, 25, 32, 38]. In this study, type III effectors were identified bioinfomatically in two plasmids (pPg2708 and pPt0893-29). The pPt0893-29 plasmid harbored the effector *hopBD1* that was also shared with the pPg2708 plasmid. Blast 2 sequence comparison revealed query coverage of 100%, with an identity of 97% (data not shown). The effector *hopBD1* and five others present in pPg2708 were characterized further in order to predict in silico their functionality (Table 3). Bioinformatic predictions based on the presence of signal peptide, transmembrane domain, and the detection of *hrp*-boxes (*hrp*-boxes are *cis*-elements shared in the promoter regions of genes encoding T3SS and type III effectors) within promoter sequences showed that five of the six putative type III effectors predicted originally in pPG2708, could be functional and thus, play an important role in virulence. In addition, the vast amount of hypothetical proteins present on the different plasmids were analyzed to determine their putative role as effectors of the T3SS (Table 4). Bioinformatic predictions based on the presence of signal peptide, transmembrane domain, and amino acid sequences using the Effective T3 tool, suggested that at least 8 hypothetical proteins could potentially function as type III effectors. One hypothetical protein was found in pPs0170, one in pPs7B44, and three in both pPg2708 and pPt0893-29 (Table 4).

**Table 3:** Bioinformatic predictions of main characteristics of putative type III effector proteins found in pPg2708 plasmid

Putative type III effectors	Size (aa)	Signal peptide	T. domains^a^	Hrp-box^b^
AvrD1	311	32–44	ND	ggaaccaaatccgtcccaaaggccacaca
AvrRps4	228	ND^c^	ND	ND
HopAU1	731	30–42	ND	ggaaccctcctgtgattttcgaacactca
HopBD1	300	110–122	ND	ggaaccgatcgaggggttctgaccacata
HopD1	713	45–57	ND	ggaacccaagagcccttgcgaccacaca
HopH1	218	12–24	ND	ggaactatcctcccacacgaagccactta

^a^ Transmembrane domains

^b^ Hrp-box is a cis promoter element recognized by the alternate sigma factor HrpL and is associated mainly with genes encoded in the type III secretion system of plant pathogenic bacteria

^c^ Non-detected

**Table 4:** Hypothetical proteins predicted as bacterial secreted effector proteins. Detection of signal peptides, transmembrane domains and sequence based prediction

H. proteins^a^	Protein size (aa)	Signal peptide (from-to)	Trans. domains (from-to)	Type III effector prediction with standard set^b^	Type III effector prediction with plant set^c^
pPs0170					
Hp11	79	1 (34–46)	ND^d^	0.99817	NP^e^
pPs7B44					
Hp6	191	1 (26–38)	ND	0.94647	NP
pPg2708					
Hp10	242	1 (55–67)	ND	0.99997	1
Hp18	160	1 (14–26)	ND	0.99922	1
Hp27	94	1 (7–19)	ND	0.99988	0.99994
pPt0893-29					
Hp4	66	1 (1–9)	ND	0.99903	0.9804
Hp14	62	1 (1–8)	ND	0.99447	NP
Hp27	140	1 (60–72)	ND	NP	0.99798

^a^ Hypothetical proteins that meet the three requirements, in all the plasmids analyzed

^b^ Standard classification module: comprises effectors of *Escherichia coli*, *Salmonella*, *Chlamydia*, *Yersinia*, and *Pseudomonas*

^c^ Plant classification module: comprises effector sequences from *Pseudomonas syringae*

^d^ *ND* Non-detected

^e^ *NP* Non-predicted

*Pseudomonas syringae* PFPs have been also demonstrated to encode other Hrp-independent virulence factors such as enzymes for phytohormone biosynthesis, which are also encoded in plasmids from other plant pathogens [25, 47]. pPt0893-29 is the unique plasmid from this study that encoded other potential virulence genes such as anthranilate synthase I and II, involved in tryptophan synthesis, levansucrase C related with the synthesis of levan, and finally a diguanylate cyclase (GGDEF) potentially involved in cyclic di-GMP synthesis, a second messenger compound that is involved in virulence of plant and opportunistic human *Pseudomonas* spp. pathogens and other plant pathogens [48–51].

### Phylogenetic analysis redefined the evolutionary history of pPT23A family plasmids

**Fig. 3:**
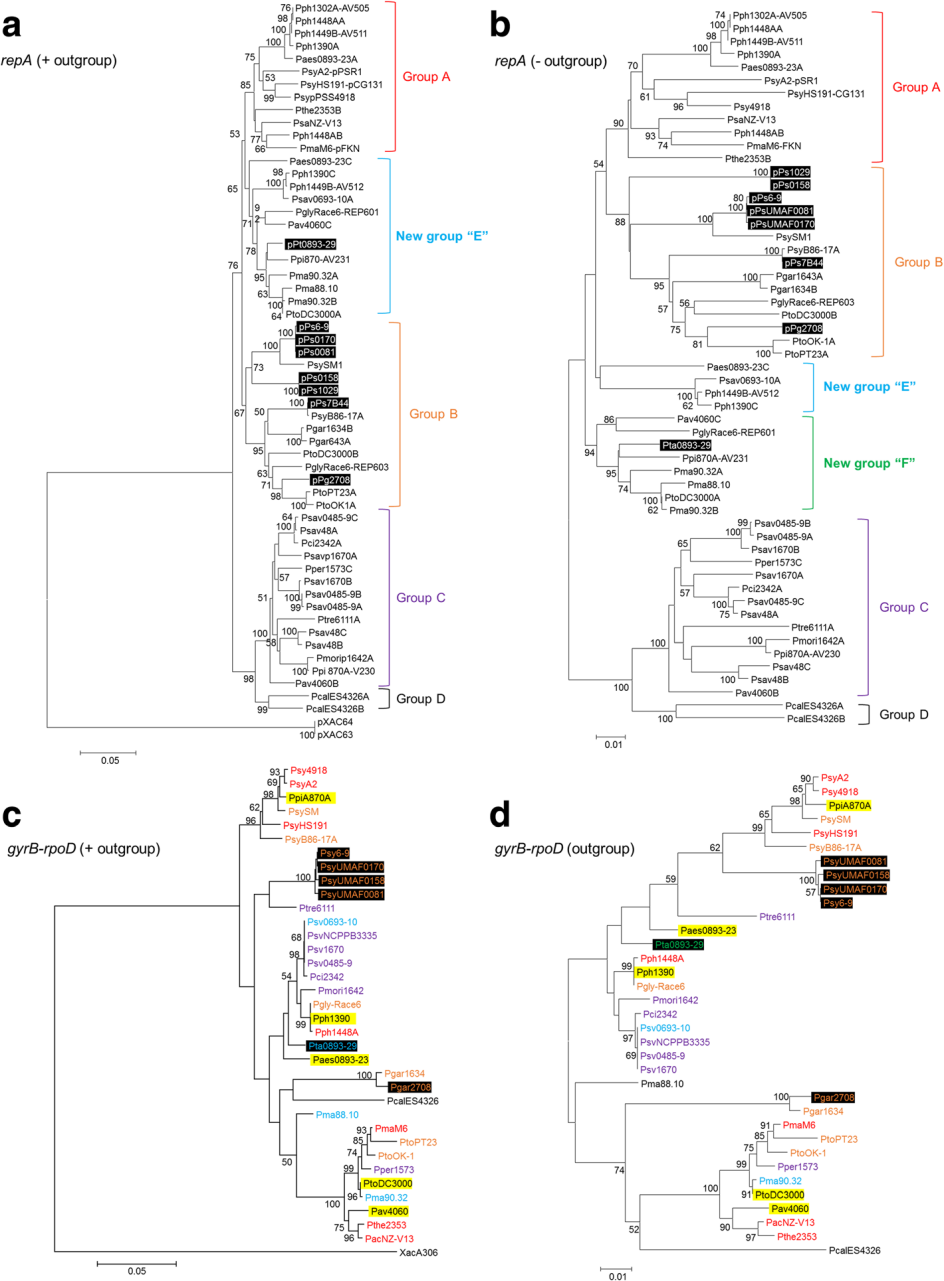
Evolutionary history of pPT23A family plasmid and their *Pseudomonas syringae* and related *Pseudomonas* hosts. **a** Phylogenetic analysis of the complete *repA* sequences of pPT23A family plasmids from *Pseudomonas syringae* and related strains, using as the outgroup the *repA* sequences of two plasmids (p33 and p64) from *Xanthomonas citri* pv. citri A306. **b** Phylogenetic analysis using the *repA* sequences from *Pseudomonas syringae* plasmids, not including in this tree the outgroup plasmids. **c** Phylogeny of *Pseudomonas syringae* and related strains harboring plasmids from the pPT23A family was constructed based on the analysis of two concatenated housekeeping genes (*gyrB* and *rpoD*), using as the outgroup the *gyrB-rpoD* sequences from *Xanthomonas citri* pv. citri A306. **d** Phylogenetic analysis using the concatenated housekeeping genes (*gyrB* and *rpoD*) from *Pseudomonas syringae* and related strains, not including in this tree the outgroup strain. The neighbor-joining tree was constructed by MEGA 5 using the Jukes-Cantor model. Percent bootstrap values of more than 50% (1000 repetitions) are shown at the nodes. The topology of the different trees, was identical by using the minimum evolution and maximum parsimony methods. *Gray boxes*: the outgroup plasmids and strain. *Black boxes*: plasmids sequenced in this study. *Yellow Boxes*: corresponding with *P. syringae* strains that harbor two pPT23A plasmids, with each plasmid belonging to a different evolutionary cluster

In order to unravel the evolutionary history of the *P. syringae* PFPs, a phylogenetic analysis using 47 *repA* gene sequences from different *P. syringae* plasmids present in the NCBI database and the *repA* sequences from the eight plasmids sequenced in this study was performed. This phylogenetic analysis was conducted using two different methods: 1) using as the outgroup the *repA* sequences of two plasmids from *Xanthomonas citri* pv. citri A306 (Fig. 3a), and 2) not using an outgroup (Fig. 3b). Phylogenetic analysis using the *repA* outgroup sequences revealed the presence of four evolutionary groups as previously described (Groups A, B, C and D; [26]), and the presence of a new group of plasmids designated as group “E” that was phylogenetically closely related with group A (Fig. 3a). This new group E, was comprised of plasmids previously characterized in group A; pPt0893-29 sequenced in this study was also included in this new group (highlighted with a black box). The remaining seven plasmids sequenced in this study were grouped together, forming part of the already known group B (Fig. 3a, highlighted with a black box). Phylogenetic analysis carried out without the use of the outgroup sequences, showed the presence of two new groups “E” and “F”, that were basically composed of the plasmids belonging to the new group E described above (Fig. 3b), although the bootstrap value for the new group E was lower than 50. In addition, these two new groups were more distantly related with group A of plasmids, in comparison with the previous phylogeny obtained using the outgroup sequences.

The trees generated with the *repA* sequences were then compared to those derived from a multilocus sequence analysis using partial sequences of *gyrB* and *rpoD* genes obtained from the database for some of the *P. syringae* strains included in the *repA* phylogeny, in order to evaluate the association of specific plasmids with specific host chromosomal genotypes. The phylogenetic groups generated were practically identical irrespective of using or not using outgroup sequences (Fig. 3c and d). Following the color scale, we could observe how the evolutionary history of PFPs and their bacterial hosts was incongruent. The *P. syringae* pv. syringae strains isolated from mango (UMAF0081, 0158 and 0170) along with the strain isolated from sweet cherry, formed a unique cluster. *P. syringae* pv. garcae 2708, grouped together with the other pv. garcae strain included in this study, and *P. syringae* pv. tabaci 0893–29 was grouped together with different pathovars of *P. syringae* (pv. aesculi, pv. phaseolicola, pv. glycinea, etc.).

**Fig. 4:**
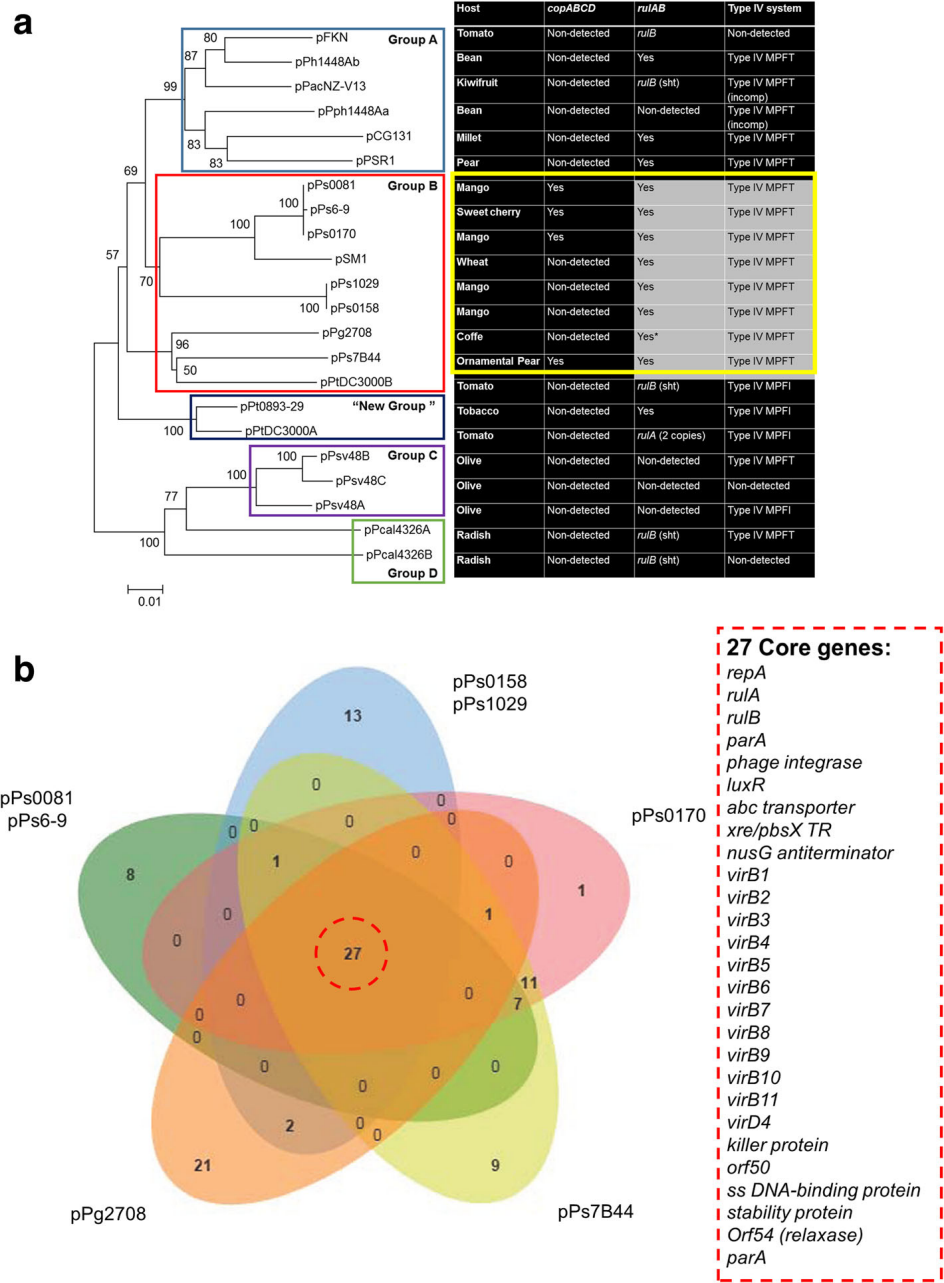
Phylogenetic relationships predict a new sub cluster into the plasmid group B. **a** Phylogenetic analysis using the *repA* sequences only from plasmids, whose their complete sequences are available in the database. The neighbor-joining tree was constructed by MEGA 5 using the Jukes-Cantor model. Bootstrap values (1000 repetitions) are shown at the nodes. The plant host of isolation and the presence of some characteristic genes of the pPT23A family plasmids (such as *copABCD* and *rulAB* operon, and Type IV secretion system) are represented in the right columns beside each plasmid name. sht: sequence shorter than expected; imcomp: incomplete; *: *rulA* and *rulB* genes located at different positions and orientations. **b** Genomic diversity of the sub cluster of plasmids. Each plasmid is represented by an oval. The number of orthologous protein-coding genes shared by all plasmids (i. e., the core genes) is in the centre. Overlapping regions show the number of coding sequences (CDS) conserved only within the specified plasmids. Numbers in the non-overlapping portions of each oval show the number of CDS unique to each plasmid. Hypothetical proteins were not taking into account to this analysis

In order to gain a greater understanding with respect to the evolutionary history of the pPT23A family, and how these plasmids could play a major role in *P. syringae* biology, the phylogenetic distribution based on the *repA* sequences (of those plasmids with complete sequences available) and its relationship with the host of isolation, and with other important genetic features of plasmids of this family (copper and UV resistance, and the presence of type IV secretion system) was analyzed (Fig. 4a). A total of 22 *repA* sequences were used for this purpose. From this analysis, we observed the presence of the original four groups, and the presence of one new group formed by pPtDC3000A from *P. syringae* pv. tomato, and by the pPt0893-29 plasmid from the pv. tabaci, that was sequenced in this study. The rest of the plasmids sequenced in this study were placed in group B, and all of them harbored the *rulAB* operon and the type IV MPF_T_ secretion system. To decipher if there were more genes shared by seven plasmids sequenced in this study that belonged to group B, an analysis of the core genome was carried out by using a Venn diagram analysis (Fig. 4b). This analysis revealed that this sub-group of plasmids share 27 core genes related mainly with maintenance and mobilization functions.

**Fig. 5:**
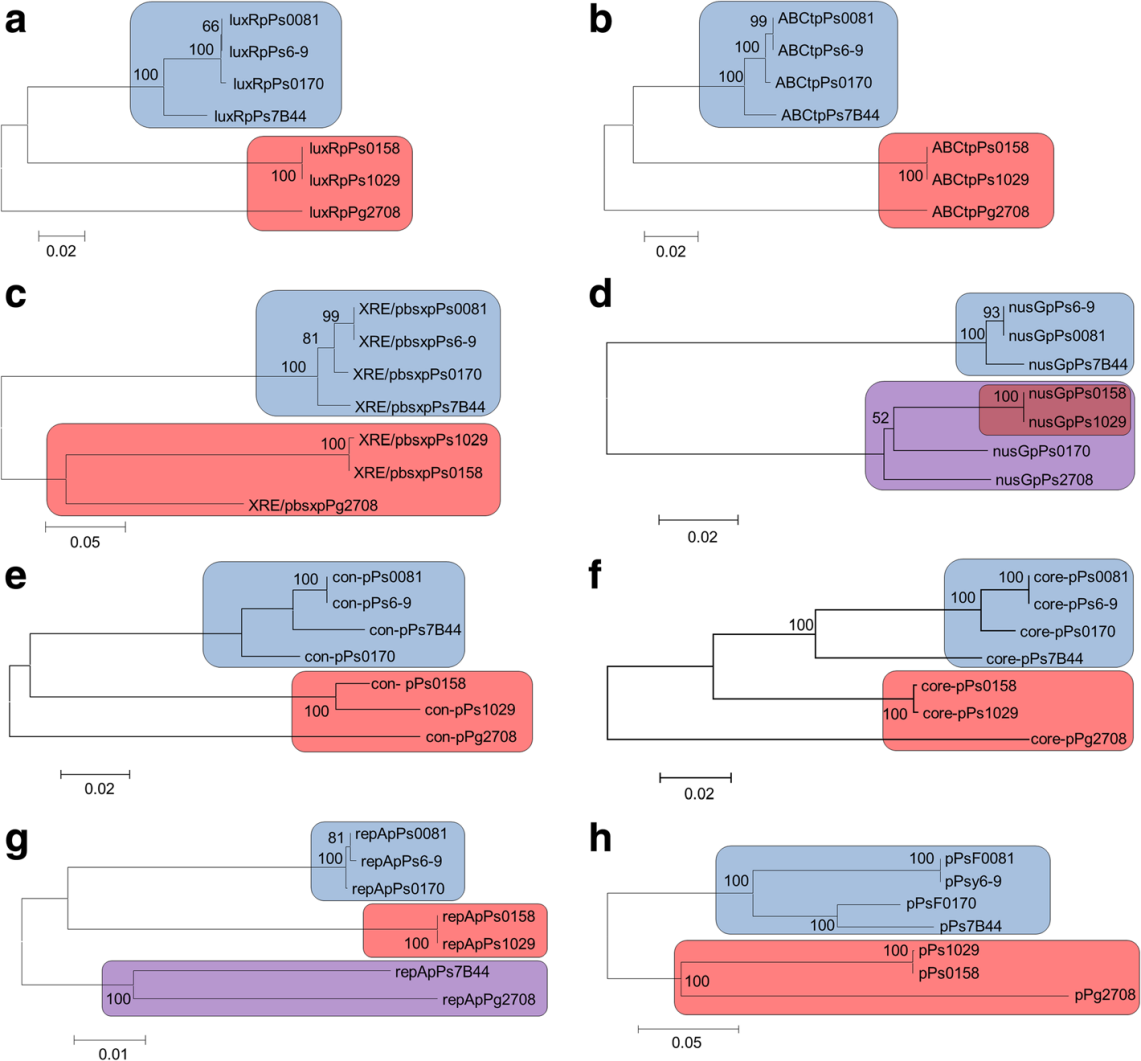
Phylogenetic distribution of putative signalling-like genes related with the conjugation system. The *luxR* transcriptional regulator, *abc* transporter, *XRE/pbsX* transcriptional regulator and *nusG* transcriptional regulator-termination nucleotide sequences from the 7 plasmids of this study that harbor the type IV MPF_T_ secretion system, were used independently (**a**, **b**, **c** and **d**) and concatenated (**e**) to carry out a phylogenetic analysis. In addition, the phylogeny of these plasmids was carried out using the core genes obtained for these plasmids in this study (27 genes) (**f**), using the *repA* sequences (**g**) and analyzing a concatenated dataset containing 27 core plasmid sequences (**h**). The neighbor-joining tree using the Jukes-Cantor model was generated by MEGA 5. Bootstrap values (1000 repetitions) are show on the branches. *Blue box*: copper resistance plasmids. *Red box*: non-copper resistance plasmids. *Purple box*: copper resistance and non-resistance plasmids

Possibly the most interesting genes regarding plasmid-specific functions revealed by the core genome analysis could be related with the induction or repression of the conjugation system, and were present in seven of eight plasmids sequenced in this study. These genes included a *luxR* transcriptional regulator, an *ABC* transporter, a *XRE*/*pbsx* transcriptional regulator and a *nusG* transcriptional regulator-termination protein; all of them located upstream of the type IV MPF_T_ secretion system. Interestingly, the *XRE*/*pbsx* transcriptional regulator and the *nusG* transcriptional regulator-termination protein are also present upstream of the type IV MPF_I_ secretion system of pPt0893-23. A phylogenetic analysis was conducted for these genes independently (Fig. 4a, b, c and d), concatenated (Fig. 5e), and also using 27 concatenated core genes (Fig. 5f) and the complete plasmid sequences (Fig. 5h), from the seven plasmids that harbored the type IV MPF_T_ secretion system. The distribution showed for the different phylogenetic analysis was similar, and grouped together the plasmids that harbored copper resistance genes (blue); the plasmids that did not encode copper resistance were contained in two separate groups (red). These analyses held for each gene analyzed except *nusG* (Fig. 5d). Also, this phylogenetic distribution was altered, when the *repA* phylogeny of the seven plasmids was used (Fig. 5g). Three different groups were observed, plasmids encoding copper resistance genes (blue), plasmids not harboring copper resistance genes (red), and another group with both copper resistance and copper sensitive plasmids (purple). This altered phylogenetic distribution was not observed when the analysis was conducted using the 27 core genes and the complete plasmid sequences (Fig. 5f and h).

## Discussion

We report the complete closed sequence and comparative genomics analysis of eight PFPs from the plant pathogen *P. syringae*. As previously reported, PFPs share the major replication gene *repA* [15, 19]; this major replication gene is the only gene currently known to be distributed among all plasmids of the pPT23A family. The pPT23A plasmid family is ubiquitous but not universal within the species *P. syringae*, as there have been strains isolated that do not contain any plasmids. However, most *P. syringae* strains studied contain one or multiple PFPs; a unique feature of the pPT23A family is the coexistence of two to several PFPs within individual *P. syringae* strains. The origin of multiple PFPs within individuals may be through incremental acquisition of plasmids or through plasmid duplication events [25, 26]. The compatibility of multiple PFPs within the same host cell was originally hypothesized to be due to alterations in amino acid sequence at the C-terminus of RepA [15]. However, more recent evidence has suggested that a putative control region sequence consisting of stem-loop structures upstream of *repA* enables compatibility of PFPs if the sequence differs by only a few nucleotides [52].

A previous phylogenetic analysis of PFPs indicated incongruence between PFP phylogeny and host *P. syringae* pathovar phylogeny [26]. Those results suggested that horizontal transfer had played a role in the current observed distribution of PFPs within *P. syringae*, and that specific plasmid:pathovar relationships did not exist. Experiments performed in vitro have demonstrated conjugative transfer for PFPs, including some instances of interpathovar transfer [23, 28, 45]. Our current results including the eight plasmids sequenced in this study and additional recent sequences from other studies confirmed this finding of incongruence of PFPs and *P. syringae* host pathovars. Furthermore, a phylogenetic analysis using a concatenated set of 27 conserved core pPFP sequences also supported the incongruence of PFPs and individual *P. syringae* pathovars.

The major contribution of PFPs to the ecological fitness and pathogenesis of *P. syringae* is through carriage of accessory genes. For example, PFPs may encode any of the following genes including the *rulAB* genes that confer resistance to ultraviolet radiation, type III effector genes, toxin biosynthesis genes, indole acetic acid biosynthesis genes, levansucrase, and agriculturally-important resistance genes to copper or streptomycin [27–29]. The carriage of specific accessory genes on PFPs can be influenced by selection acting at the *P. syringae* species level, with carriage of genes such as *rulAB* [44]. Other genes such as type III effector genes are likely selected at the pathovar level (i.e., important to a specific *P. syringae*-host interaction), and still others at the population level (i.e., an intrapathovar distribution important in a specific environment). For example, the plasmids pPs6-9 and pPS7B44 (Fig. 1) were isolated from *P. syringae* pv. syringae strains under selection from copper bactericide use on different woody hosts from different regions of the United States. Both of the plasmids encode highly similar backbones with the only major difference being the type of copper resistance determinant acquired which likely reflects the availability of genes within the respective communities.

The PFPs that have been sequenced from *P. syringae* pv. syringae strains in this and other studies have encoded few or no genes encoding type III effectors with potential direct linkages to *P. syringae*-host interactions [22, 34, 35]. This is in contrast to other PFPs, such as pPg2708 and pPt0893-29 in this study, and others that encode multiple type III effectors and additional putative virulence genes [25, 38]. We hypothesize that this is a reflection of the number of effectors required to cause disease and suppress host resistance responses in particular pathosystems; for example, genome sequence analysis of different *P. syringae* pv. syringae strains has shown a relatively small number of type III effectors encoded by these strains [53] compared to other pathovars. Continued acquisition of type III effectors by PFPs may be important in the pathogen-host “arms race” positively affecting virulence in other pathovars encoding large effector repertoires. The plasmid pPg2708 from this study presents the *rulB* gene nicked, and an insertion of 36.5 Kbp of DNA was found, and, carrying among others, 4 type III effectors. The role of *rulB* gene as a hotspot for site specific insertion of integron-like elements (ILEs) and other putative mobile elements that harbor T3SS effectors among other genes has been previously described [54, 55].

We detected large regions from the chromosomally-located GI6 genomic island of *P. syringae* pv. syringae B728a on two of the PFPs (pPs0170 and pPs7B44) sequenced in this study. This provides further retrospective evidence of the movement of genomic island regions between plasmid and chromosome in *P. syringae*. The GI6 genomic island shares extensive similarity to the PPHGI-1 genomic island of *P. syringae* pv. phaseolicola 1302A, a 106-kb genomic island that can excise from the chromosome when strain 1302A is inoculated onto a resistant bean cultivar [56]; however, the copper and arsenic-resistance determinants found on the plasmids sequenced in this study are not present in the PPHGI-1 island. The integration of plasmids into and excision of plasmids from the *P. syringae* chromosome was first demonstrated 35 years ago [57, 58]. Later, the PFP pFKN was shown to integrate into the chromosome of *P. syringae* pv. maculicola via recombination between alleles of the T3SS effector *avrpPhE* [31], and the PFP pAV505 of *P. syringae* pv. phaseolicola 1302A and two other native plasmids were presumably integrated into the chromosome of the host strain after passage through a resistant bean cultivar [59]. In many cases, these intragenome plasmid recombination or excision events appear to occur as a consequence of host recognition/avoidance scenarios [60]. However, the recombination and relocation of genomic island sequences such as the copper and arsenic-resistance determinants to pPs0170 and pPs7B44 also suggests that other ecological stress selection can play a role in gene movement between plasmids and chromosomes.

The carriage of large plasmids imposes a fitness cost on the host cell that must be ameliorated to enable plasmid maintenance. This coevolutionary process can be mediated through any of several mechanisms including deletion of nonessential sequences from the plasmid, alterations (typically reductions) in plasmid gene expression, and alterations in chromosomal gene expression (reviewed in [61]). The importance of positive selection for plasmid-encoded genes, such as antibiotic-resistance genes, in conjunction with other coevolutionary mechanisms also plays a critical role in plasmid persistence [62]. Conjugative plasmids exhibit a modular structure consisting of discrete gene regions that are clustered into functional groups [12]. These functional groups or modules include replication, stability, propagation, and adaptation with the propagation module including genes functioning in conjugative transfer and the adaptation module including ecologically-important genes [63]. Coordination and co-regulation of genes within these functional modules is also important and contribute to an overall “plasmid survival kit” [63].

Our sequence analysis of PFP genomes in this study illustrated a modular structure of these plasmids (Fig. 1) with replication, stability, propagation, and adaptation modules all present. The ubiquitous nature of PFPs within *P. syringae* suggests that this plasmid family has successfully coevolved with the *P. syringae* species, however, few studies have assessed the effect of PFPs on the corresponding fitness of the *P. syringae* host [45, 64]. Co-regulation of the plasmid-selfish genes (replication, stability, propagation modules) likely contributes to a reduction of deleterious effects of PFPs on their *P. syringae* hosts. Similarities in gene content and synteny of these modules in the plasmids examined in this study and in previous analyses of PFPs genomes [15, 21, 41], suggest that the modules that contribute to the plasmid backbone are strongly conserved.

There is one curious distinction in gene content among the plasmid backbone genes present on PFPs and that involves the composition of the propagation module specifically with regards to conjugative transfer related genes encoding a type IV secretion system (T4SS). There are two known suites of conjugative transfer genes on PFPs, the type IV MPF_T_-T4SS (VirB-VirD4 conjugative system) and the type IV MPF_I_-T4SS (*tra* conjugative system) [21]. In a previous macroarray study of 31 PFPs from 12 *P. syringae* pathovars, we identified 12 PFPs that hybridized to most or all of the type IV MPF_T_-T4SS genes and 10 PFPs that hybridized to all 21 genes of the type IV MPF_I_-T4SS genes [21]. The PFPs that encoded a type IV MPF_T_-T4SS were distributed among *P. syringae* pathovars in genomospecies I, II, III, and IV, and PFPs that encoded a type IV MPF_I_-T4SS were distributed among *P. syringae* pathovars in genomospecies II, III, and IV [21]. Only a few other PFPs were shown to either lack a T4SS or to only hybridize to a few T4SS genes [21]. As shown in the plasmids maps in Fig. 1, the genetic location of either of the type IV MPF_T_ or type IV MPF_I_ T4SSs is very similar, and upstream of the *gntR* transcriptional regulator, *repA*, and the *rulAB* genes, suggesting that both of these T4SS gene sets have been acquired into an existing PFP backbone. In addition, it is not yet clear if carriage of either of the type IV MPF_T_ or type IV MPF_I_-T4SSs is associated with carriage of specific accessory genes including type III effector genes.

Finally, analysis of accessory gene carriage among sequenced plasmids common to gram-negative plant pathogens such as *P. syringae*, *Erwinia amylovora*, and *Xanthomonas campestris* [15, 65–68] shows a predominance of known and putative virulence determinants and less numbers of genes that may generally enhance fitness in the plant environment. This is in spite of the observation that these plant pathogens do spend parts of their life cycles growing on plant surfaces either on leaves or flowers. Analysis of accessory gene carriage on plasmids isolated from non-pathogenic plant-associated bacteria has shown differing suites of genes compared to those found on plasmids from plant pathogens. For example, pA506, a recently-sequenced plasmid from the plant epiphyte *P. fluorescens* A506, is very similar to PFPs except it does not encode a *repA* gene or other replication initiation protein [69]. However, although pA506 encodes similar genes in the propagation and stability modules to PFPs and does encode *rulAB*, there are few other similarities in the accessory gene module [69]. Likewise the pQBR plasmid family which is distributed among bacterial strains isolated from the sugar beet phyllosphere, does not appear to share accessory genes with any other PFPs [70]. Thus, plant pathogens such as *P. syringae* harbor plasmids that encode accessory genes with a plant pathogen “signature” that is important for host-pathogen interactions and/or is selected in response to bactericide usage in agriculture. This suggests in turn that other genes, such as specific epiphytic fitness determinants critical for in planta growth in these pathogens, are maintained as indispensible in chromosomal locations freeing the plasmids to carry genes that, though still dispensible overall to the host bacterium, provide a selective advantage that is strong enough both to maintain the plasmid within the bacterial pathogen population and to maintain the bacterial pathogen populations on their respective plant hosts.

## Conclusions

Our sequence analysis revealed that PFPs from *P. syringae* encode suites of accessory genes that are selected at species (universal distribution), pathovar (interpathovar distribution), and population levels (intrapathovar distribution), and contribute to ecological and pathogenic fitness. Results from phylogenetic analyses supported the incongruence of PFPs and individual *P. syringae* pathovars, suggesting the importance of horizontal transfer in affecting the current distribution of PFPs in the *P. syringae* species. The conservation of type IV secretion systems encoding conjugation functions also presumably contributes to the distribution of these plasmids within *P. syringae* populations.

## Methods

### *Pseudomonas syringae* strains and their native plasmids

*Pseudomonas syringae* strains used in this study are summarized in Table 1. The main characteristics from the different native plasmids isolated from the different *P. syringae* strains and sequenced in this work are described in Table 2. *Pseudomonas syringae* strains were routinely grown at 28 °C using Lysogeny (LB) broth and agar medium.

### Plasmid DNA Isolation and purification

Large-scale plasmid DNA extractions were conducted using 1000 mL LB broth cultures for each strain according to a modified Maxi-preparations (maxi-prep) alkaline lysis method [66, 67]. The sizes of the different plasmids were estimated based on a control reference strain, *P. syringae* pv. tomato PT23 that harbors four plasmids (pPT23A, pPT23B, pPT23C and pPT23D that are 100-Kb, 83-Kb, 65-Kb and 36-Kb in size, respectively) [71]. Subsequently, plasmid DNA purification was carried out by equilibrium centrifugation in cesium chloride-ethidium bromide gradients (CsCl-EtBr) gradients [72]. Briefly, each plasmid maxi-prep was resuspended in 8 mL of TE buffer (pH 8.0), and 8.8 g of CsCl and 800 μL of EtBr (10 mg/mL) were added. These gradient preps were subjected to ultracentrifugation (16 h, 194,000 × *g*, 20 °C), and then the plasmid DNA was collected using a hypodermic needle and a disposable syringe. Then, the EtBr and CsCl were removed from the plasmid DNA extraction [73]. Finally, the plasmid DNA was precipitated and dissolved in 0.5 mL of TE buffer (pH 8.0).

### Plasmids sequencing and annotation

The complete plasmid DNA sequences were obtained using Roche 454 GS (FLX procedure) platform, and were carried out at the Genomics Technology Support Facility, Michigan State University. DNA sequences for each plasmid were assembled using Newbler Ver2.0 (Roche 454 Life Sciences). In order to validate the sequencing method used in this study, the sequencing of one of the plasmids was also carried out at the Beijing Genomics Institute (BGI-HK) using Illumina HiSeq 2500 system. The plasmid selected for this purpose was the plasmid from *P. syringae* pv. syringae UMAF0158 that has been recently published [74]. Comparative sequence analysis of this plasmid sequenced by different methods was carried out using BLAST 2 Sequences tool from NCBI [75].

The complete sequence from the eight different native plasmids were then placed in the same orientation, taking as nucleotide +1, the nucleotide +1 of the *repA* gene. The plasmids were automatically annotated using Rapid Annotations using Subsystems Technology (RAST) [76]. Subsequently, they were manually refined using different tools such as are BLASTx [77] and the graphical analysis tool ORF Finder (http://www.ncbi.nlm.nih.gov/gorf/gorf.html). The search of open reading frames (ORFs) that were detected by BLASTx but not by ORF Finder, including the ORFs with alternative start codons (gtg and ttg) was carried out using Redasoft Visual Cloning 3.2. This software was also used for the construction of the genetic plasmid maps [23]. The last revision of plasmid sequence annotation was done in November 2016.

### Molecular biology techniques

Polymerase chain reaction (PCR) method using specific primers designed on the eight different plasmid DNA sequences (Table S1) was carried out in order to test if the sequencing method used in this study generated correctly closed circular DNA. The PCR was performed between the start and the end of each plasmid using the raw DNA sequences provided by the Genomics Technology Support Facility, Michigan State University.

For this purpose, genomic DNA was extracted by using the DNeasy tissue kit (QIAGEN Inc., USA) according to the manufacturer’s instructions. Furthermore, plasmid DNA mini-preparations were performed using 1.5 mL of an LB broth overnight culture following a modified alkaline lysis method [71]. DNA concentrations and quality from both were determined using NanoDrop ND-1000 (NanoDrop Technologies) spectrophotometer and by agarose gel electrophoresis.

### Bioinformatic analysis

#### Phylogenetic studies

A phylogenetic analysis using the *repA* gene sequences of the eight plasmids sequenced in this study, and also including other *repA* sequences present in the NCBI database that belong to other plasmids from the same family (pPT23A) was conducted. In addition, an evolutionary history study based on the analysis of the *gyB* and *rpoD* housekeeping genes of the *Pseudomonas syringae* and related *Pseudomonas* whose harbor the pPT23A plasmids was carried out. The different DNA sequences were alignment using Clustal W2 [78] and the different phylogenetic trees were generated using MEGA 5 [79] with neighbor-joining, Jukes-Cantor model, minimum evolution, and the option of complete deletion to eliminate positions containing gaps. Confidence levels of the branching points were determined using 1000 bootstrap replicates.

#### Comparative plasmid sequence (plasmidomic) analysis

The eight native plasmids sequenced in this study were aligned using “MAUVE: Multiple Genome Alignment” to analyze the plasmids synteny [80]. In addition, MAUVE allows efficient construction of multiple alignments in the presence of large-scale evolutionary events such as rearrangements and inversions. The eight native plasmids were compared using the Venn diagram analysis software “jvenn” [81] in order to define the core genes.

In addition, the pPg2708 plasmid was analyzed in order to characterize the promoters of the *rulAB* genes that are not forming an operon in this plasmid. The promoter prediction was performed using BPROM from SoftBerry (http://www.softberry.com, Mount Kisco, NY, USA). The Shine-Dalgarno (SD) sequences were also defined according to Ma et al. [82]. The search of the *hrp* box promoter sequences for the different type III effectors harbored by the pPg2708 plasmid was performed based on the consensus sequence 5′-GGAACC-N15-16-CCACNNA-3′ [83].

#### Unraveling putative encoding-Type III effector genes

All the hypothetical proteins found in each native plasmid sequenced in this study, were analyzed to determine their possible role in virulence as effector proteins of the T3SS. Signal peptide cleavage sites were predicted using the SignalP 4.1 server [84, 85] and Sigcleave whose are accessible at http://www.cbs.dtu.dk/services/SignalP/ and http://emboss.bioinformatics.nl/cgi-bin/emboss/sigcleave respectively. Prediction of transmembrane helices domains in proteins was performed using the TMHMM Server v. 2.0 [86], which is accessible at http://www.cbs.dtu.dk/services/TMHMM/. Finally, a prediction of secreted proteins based on amino acid sequences was carried out using Effective T3 [87], which is accessible at http://www.effectors.org/news/new-effectivet3-model. Two different modules were used: a standard classification module that comprises effectors of *Escherichia coli*, *Salmonella*, *Chlamydia*, *Yersinia*, and *Pseudomonas* (opportunistic human and animal pathogens), and plant classification module that comprises effector sequences from *P. syringae* (plant pathogen). In both cases, a cut-off value of 0.9 in a sensitive setting was selected to carry out the analysis.

### Nucleotide sequence accession numbers

The complete plasmid sequences obtained in this study are deposited at NCBI under the accession numbers: KY362366 (pPt0893-29), KY362367 (pPg2708), KY362368 (pPs0081), KY362369 (pPs 6–9), KY362370 (pPs0158), KY362371 (pPs1029), KY362372 (pPs0170) and KY362373 (pPs7B44). GenBank accession numbers for sequences of the chromosomal gene *gyrB* are as follows: DQ072672.1 to DQ072693.1, AB016387.1, CM001986.1, JX867861.1, JX867909.1, JX867863.1, JX867862.1, CM001834.1, AB016323.1, CP000058.1, AY610777.1, AY610778.1, NC_004578.1, NZ_CP011972.2 and NZ_CP006857.1. GenBank accession numbers for sequence of the chromosomal gene *rpoD* are as follows: DQ072694.1 to DQ072715.1, AB016388.1, CM001986.1, JX867782.1, JX867829.1, JX867784.1, JX867783.1, CM001834.1, AB016324.1, CP000058.1, AY610897.1, AY610898.1, NC_004578.1, NZ_CP011972.2 and NZ_CP006857.1. GenBank accession numbers for sequence of the plasmid replication gene *repA* are as follows: DQ072652 to DQ072671, AY768793.1 to AY768802.1, AY768804.1 to AY768807.1, NC_004632.1, NC_004633.1, CP000059.1, CP000060.1, NC_019265.1, NC_019266.1, NC_019292.1, NC_002759.1, NC_005918.1, NC_005919.1, NC_005205.1, NZ_CP011973.1, NZ_CM001987.1, NZ_CP006855.1, NZ_CP006856.1 and including the *repA* sequences of the 8 plasmids sequenced in this study.

## Additional files

Additional file 1: Table S1. Primers used in this study. (PDF 14 kb)

Additional file 2: Figure S1. Polymerase chain reaction to test the closed plasmids DNA sequences. Amplicons of the expected size obtained using specific primers designed on the final and the beginning of the raw plasmid DNA sequences. M: molecular weight marker HyperLadder 1Kb (Bioline). GD: PCR carried out using Genomic DNA; PD: PCR carried out using Plasmid DNA. (PDF 78 kb)

Additional file 3: Table S2-S9. Predicted ORF’s in the different plasmids sequenced in this study. (PDF 488 kb)

Additional file 4: Figure S2. Plasmid sequences alignment. Pairwise alignment between the 8 different plasmid sequenced in this study, was carried out using the MAUVE software. Colored blocks: plasmid sequences that aligned to other plasmid parts, being presumably homologous without no internal rearrangement. White regions: probably specific sequence elements to a particular plasmid. Blocks below the central line of each plasmid represent sequences that aligned in the reverse complement orientation. (PDF 247 kb)

Additional file 5: Figure S3. Type IV secretion systems analysis. **a** The type IV MPF_T_ secretion system is normally encoded by 11 virB genes and the virD4 gene. Graphical map for the type IV MPF_T_ secretion system genes from the plasmids sequenced in this study, including the type IV MPF_T_ of the pPSR1 plasmid from *Pseudomonas syringae* pv. syringae A2. **b** Schematic representation of the type IV MPF_I_ secretion system genes from pPt0893-29 plasmid sequenced in this study, including the type IV MPF_I_ from pPtDC3000A from *Pseudomonas syringae* pv. tomato for their comparison. Genes with similar functions were drawn with similar colors. (PDF 33 kb)

Additional file 6: Figure S4. Plasmid map graphical comparison of pPs0170 and pPs7B44. Green dotted lines and grey box represent a sub part from the genomic island GI6 from *Pseudomonas syringae* pv. syringae B728a forming part of both plasmids. Purple arrows: Arsenic resistance genes. (PDF 112 kb)

## Abbreviations

CsCl-EtBr: Cesium chloride-ethidium bromide
PCR: Polymerase chain reaction
PFP’s: pPT23A-family plasmids
pv.: Pathovar
T3SS: Type III secretion system
