# Supplementary material for: Complete sequence and comparative genomic analysis of eight native Pseudomonas syringae plasmids belonging to the pPT23A family
Source: BMC Genomics. 2017 May 10;18:365. doi: 10.1186/s12864-017-3763-x (PMC5424326; doi:10.1186/s12864-017-3763-x)
Supplement: Supplementary file 1 — Primers used in this study. (PDF 14 kb) [file 12864_2017_3763_MOESM1_ESM.pdf]

Table S1 Primers used in this study to check the start and end of each plasmid

P. syringae	**Primer name**	**Sequence (5´-3´)**	**Amplicon size (bp)**
pv. syringae			
UMAF0081	0081F	CCTGCCGCTGTCTTCGATAC	775
	0081R	GTGGTCCGCAGCAGGATTAG	
UMAF0170	0170F	TAGTAGCCTGGCCACACTTG	813
	0170R	TGCACAAACCACCGCGATAC	
UMAF0158	0158F	CCTGATGATGGCCGCACTTG	761
	0158R	GGCAGAGCTGGCCAAATAC	
UMAF1029	1029F	CGTTTAGTCCCGCCTTATGC	862
	1029R	CCTTGCGCGTGTAAAGATCC	
6-9	6-9F	ATCAATCAGCCGGCGAGTTC	796
	6-9R	CTGCGTGCCACCAACAAAG	
7B44	7B44F	TGCGTGCGCCTCACATCTAC	767
	7B44R	TGCGCCTGAAGACGCTTTGC	
pv. garcae			
NCPPB2708	2708F	GGTAACGCGCTGCTGATAGG	748
	2708R	CCTGAAGCGGCTCTGTACAC	
pv. tabaci			
0893-29	0893F	GCACGTTCGCCGTTGAATGG	991
	0893R	TTAAGCGACGTGGCGCCTAC	
