# Supplementary figures and images for: Complete sequence and comparative genomic analysis of eight native Pseudomonas syringae plasmids belonging to the pPT23A family
Source: BMC Genomics. 2017 May 10;18:365. doi: 10.1186/s12864-017-3763-x (PMC5424326; doi:10.1186/s12864-017-3763-x)

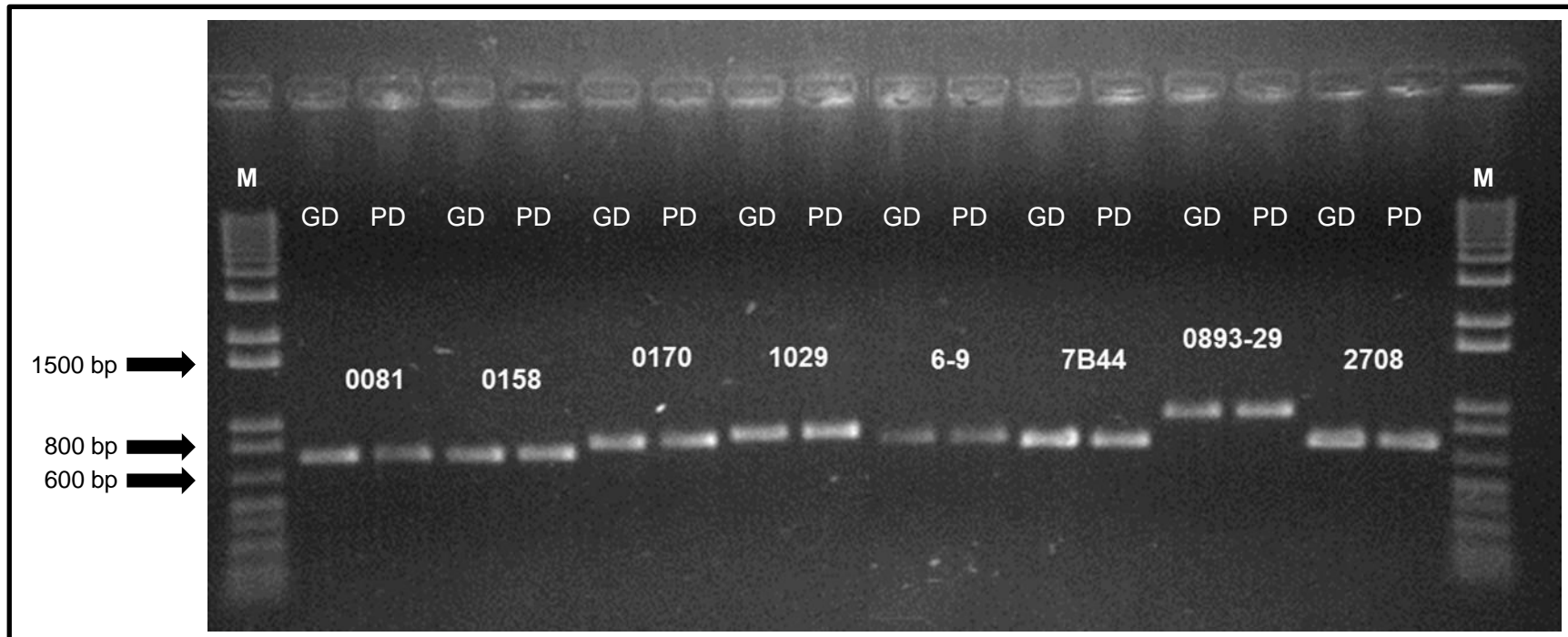

Supplement: Supplementary file 2 — Polymerase chain reaction to test the closed plasmids DNA sequences. Amplicons of the expected size obtained using specific primers designed on the final and the beginning of the raw plasmid DNA sequences. M: molecular weight marker HyperLadder 1Kb (Bioline). GD: PCR carried out using Genomic DNA; PD: PCR carried out using Plasmid DNA. (PDF 78 kb) [file 12864_2017_3763_MOESM2_ESM.pdf]

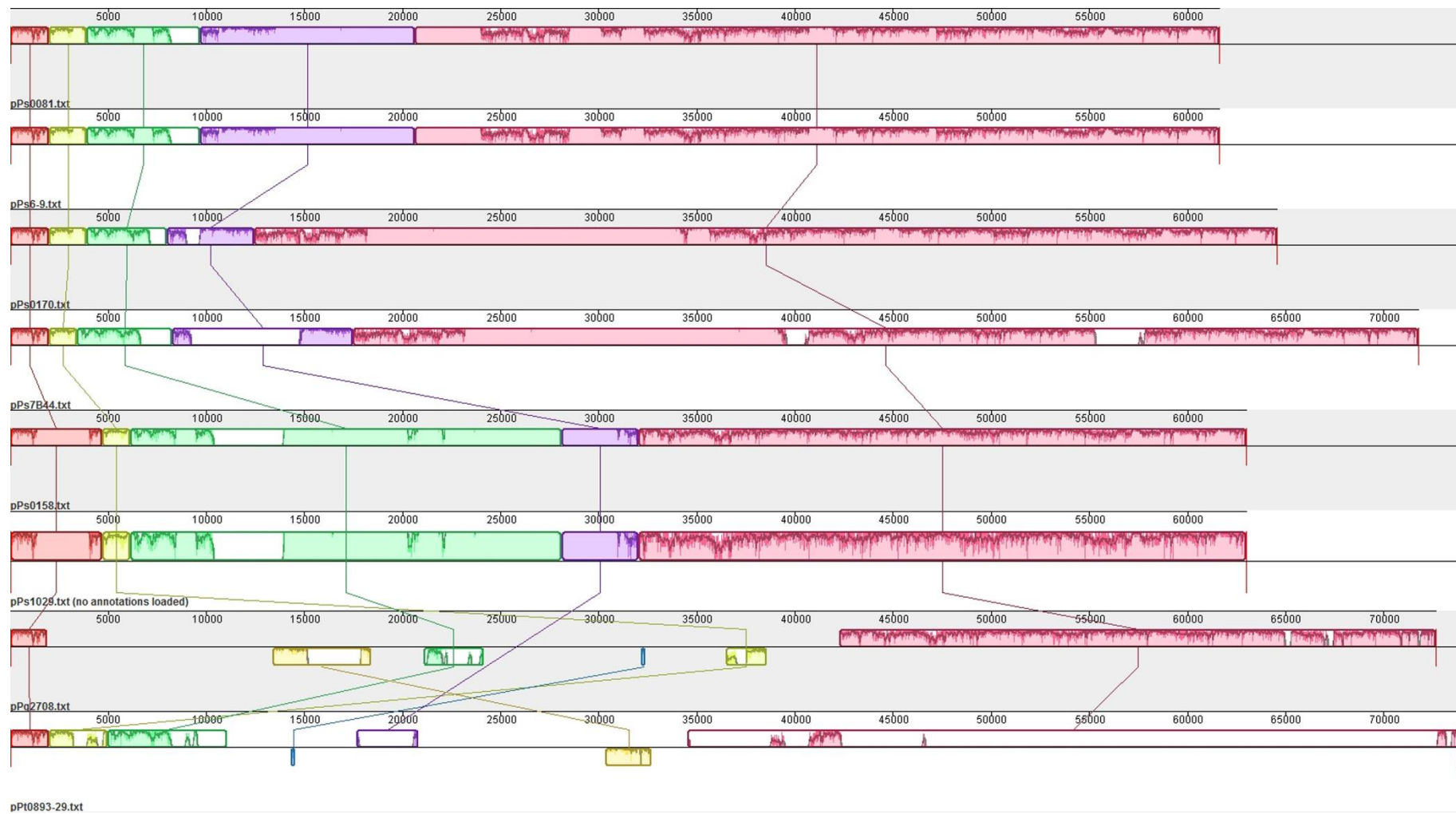

Supplement: Supplementary file 4 — Plasmid sequences alignment. Pairwise alignment between the 8 different plasmid sequenced in this study, was carried out using the MAUVE software. Colored blocks: plasmid sequences that aligned to other plasmid parts, being presumably homologous without no internal rearrangement. White regions: probably specific sequence elements to a particular plasmid. Blocks below the central line of each plasmid represent sequences that aligned in the reverse complement orientation. (PDF 247 kb) [file 12864_2017_3763_MOESM4_ESM.pdf]

a type IV MPF<sub>T</sub>

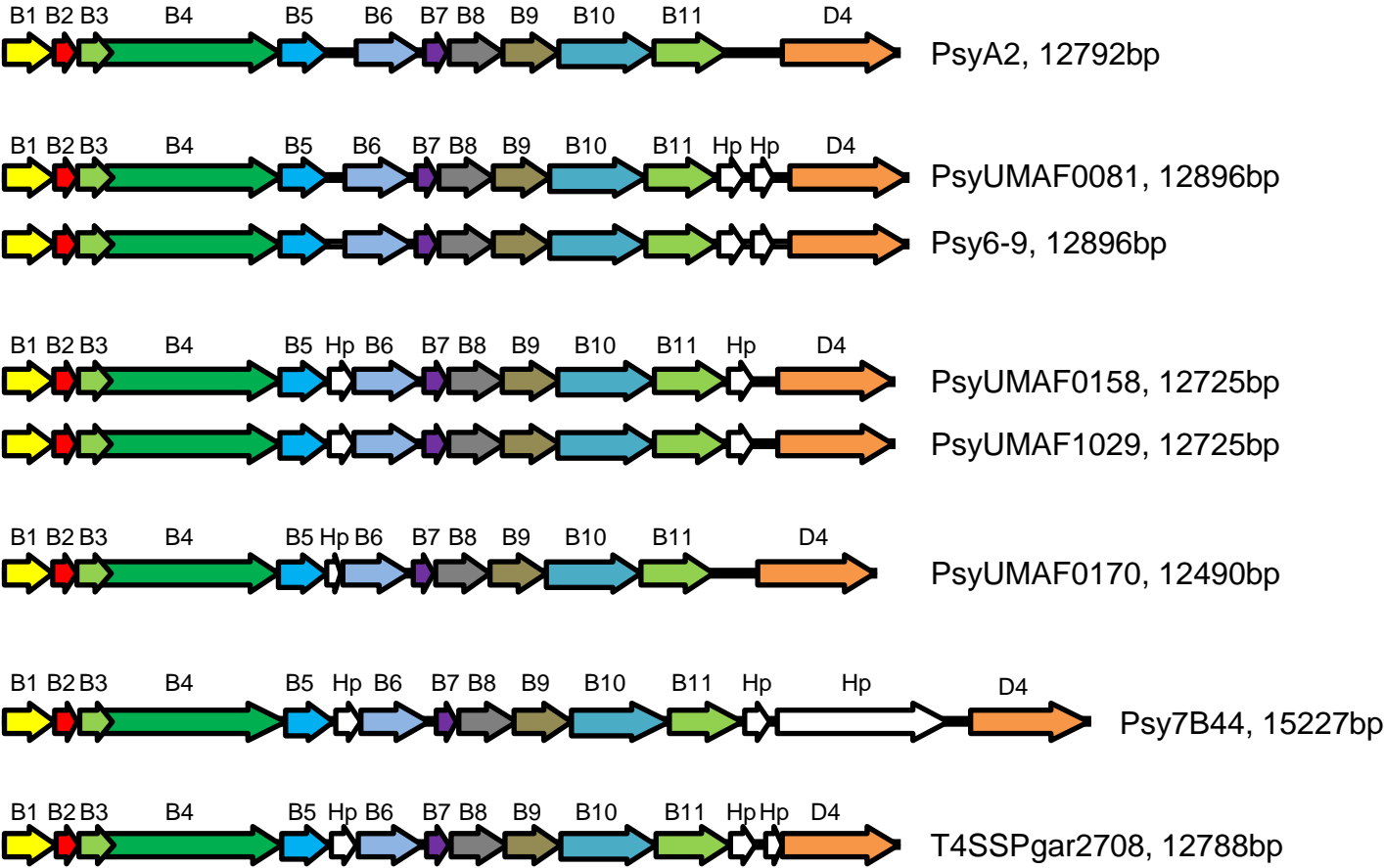

b type IV MPF<sub>I</sub>

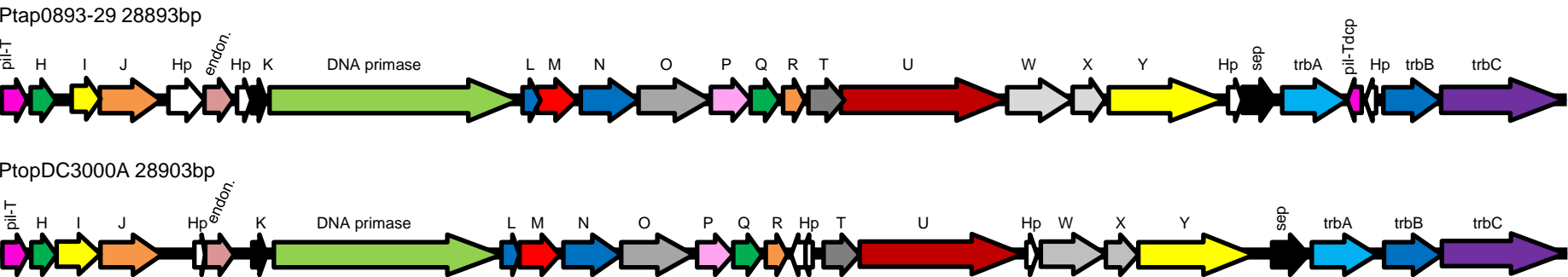

Supplement: Supplementary file 5 — Type IV secretion systems analysis. a The type IV MPFT secretion system is normally encoded by 11 virB genes and the virD4 gene. Graphical map for the type IV MPFT secretion system genes from the plasmids sequenced in this study, including the type IV MPFT of the pPSR1 plasmid from Pseudomonas syringae pv. syringae A2. b Schematic representation of the type IV MPFI secretion system genes from pPt0893-29 plasmid sequenced in this study, including the type IV MPFI from pPtDC3000A from Pseudomonas syringae pv. tomato for their comparison. Genes with similar functions were drawn with similar colors. (PDF 33 kb) [file 12864_2017_3763_MOESM5_ESM.pdf]

**pPs7B44**

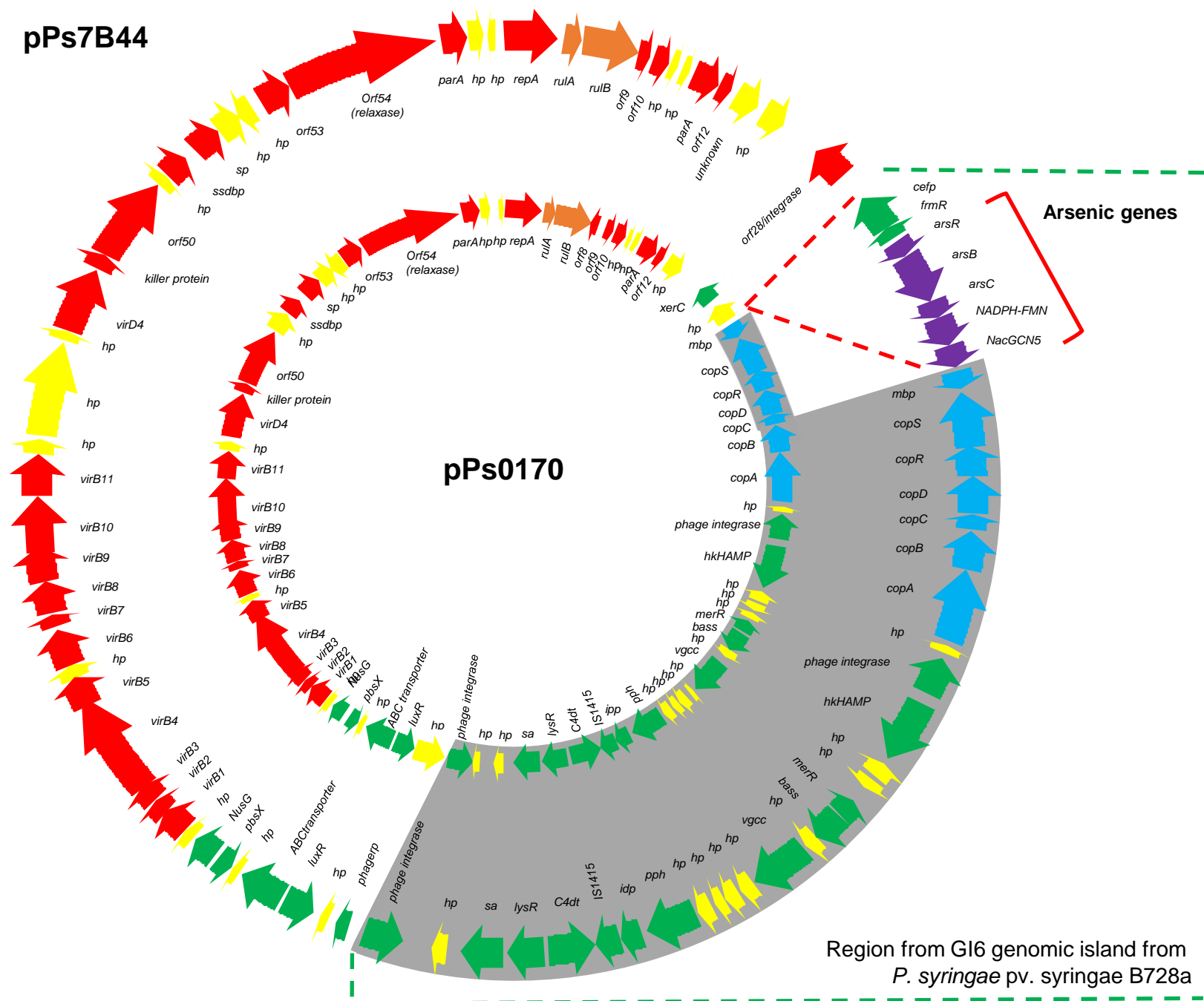

Supplement: Supplementary file 6 — Plasmid map graphical comparison of pPs0170 and pPs7B44. Green dotted lines and grey box represent a sub part from the genomic island GI6 from Pseudomonas syringae pv. syringae B728a forming part of both plasmids. Purple arrows: Arsenic resistance genes. (PDF 112 kb) [file 12864_2017_3763_MOESM6_ESM.pdf]
