# Supplementary material for: Complete sequence and comparative genomic analysis of eight native Pseudomonas syringae plasmids belonging to the pPT23A family
Source: BMC Genomics. 2017 May 10;18:365. doi: 10.1186/s12864-017-3763-x (PMC5424326; doi:10.1186/s12864-017-3763-x)
Supplement: Supplementary file 3 — Predicted ORF’s in the different plasmids sequenced in this study. (PDF 488 kb) [file 12864_2017_3763_MOESM3_ESM.pdf]

Table S2 Predicted ORFs in the *P. syringae* pv. syringae UMAF0081 plasmid

Position (5´-3´)	Size (aa)	Homology (Blast)[a]	Identity and similarity (source of homolog)	Accession No.
1-1314	437	RepA (Ps pv. tabaci ATCC 11528)	437/437 (100%), 437/437 (100%),	ZP_05639843.1
1453-1878	141	RulA (Ps pv. tabaci ATCC 11528)	141/141 (100%), 141/141 (100%)	ZP_05639842.1
1856-3154	432	RulB (Ps pv. tabaci ATCC 11528)	426/427(99%), 427/427(100%)	ZP_05639841.1
3230-3535	101	Hp (*P. syringae* group)	101/101(100%), 101/101(100%)	WP_005782530.1
3816-4103	95	Hp ALO82_200232 (Ps pv. broussonetiae)	94/95(99%), 95/95(100%)	KPW64255.1
4197-4607	136	Orf10/Hp (Ps pv. tabaci ATCC 11528)	136/136 (100%), 136/136 (100%)	WP_005782529.1
4691-4852	53	Hp (*P. syringae*)	50/53(94%), 51/53(96%)	YP_006963270.1
4945-5145	66	Hp PsyrptA_21191 (Ps pv. tabaci ATCC 11528)	66/66 (100%), 66/66 (100%)	ZP_05639838.1
5258-5908	216	Plasmid partitioning protein ParA (*P. amygdali*)	216/216(100%), 216/216(100%)	WP_003348583.1
5898-6185	95	Orf12/Hp (Ps pv. tabaci ATCC 11528)	95/95 (100%), 95/95 (100%)	ZP_05639836.1
6822-7211	129	Hp (*P. syringae* group)	129/129 (100%), 129/129 (100%)	WP_010199903.1
7390-8031	213	Hp PsyrptN_02905 (Ps pv. tomato NCPPB 1108)	213/213(100%), 213/213(100%)	ZP_07256350.1
8321-9007	228	Hp PsyrptA_21166 (Ps pv. tabaci ATCC 11528)	228/228 (100%), 228/228 (100%)	ZP_05639833.1
9023-9553	176	Hp PsyrptA_21161 (Ps pv. tabaci ATCC 11528)	176/176 (100%), 176/176 (100%)	ZP_05639832.1
9658-10617	319	Orf28/integrase (Ps pv. tabaci ATCC 11528)	319/319 (100%), 319/319 (100%)	ZP_05639831.1
10681-11361	226	Hp PsyrptA_21151/metal-binding protein (Ps pv. tabaci ATCC 11528)	226/226 (100%), 226/226 (100%)	ZP_05639830.1
11431-12810	459	Heavy metal sensor kinase, CopS (Ps pv. syringae UMAF0081)	459/459 (100%), 459/459 (100%)	AFV33241.1
12807-13493	228	Heavy metal response regulator, CopR (Ps pv. syringae UMAF0081)	228/228 (100%), 228/228 (100%)	AFV33242.1
13724-13921[b]	65	Heavy metal transport/detoxification protein/copper chaperone/CopZ (Ps pv. tabaci ATCC 11528)	65/65(100%), 65/65(100%)	gb|EGH92938.1
14079-16463	794	CopG (Ps pv. syringae UMAF0081)	794/794 (100%), 794/794 (100%)	AFV33243.1
16460-16858	132	Transcriptional regulator (*P. amygdali*) (Cu-(I) responsive trans. regulator	131/132(99%), 132/132(100%)	WP_005782518.1
16946-20092	1048	CusA (Ps pv. syringae UMAF0081)	1048/1048(100%), 1048/1048 (100%)	AFV33244.1
20089-21543	484	CusB (Ps pv. syringae UMAF0081)	484/484 (100%), 484/484 (100%)	AFV33245.1
21540-22802	420	CusC (Ps pv. syringae UMAF0081)	420/420 (100%), 420/420 (100%)	AFV33246.1
23416-23862	148	metal-binding protein (*P. amygdali* pv. tabaci str. ATCC 11528]	148/148 (100%), 148/148 (100%)	AFV33258.1
23951-24952	333	CopD (Ps pv. syringae UMAF0081)	333/333(100%), 333/333(100%)	AFV33248.1
24889-25272	127	CopC (Ps pv. syringae UMAF0081)	127/127 (100%), 127/127 (100%)	AFV33249.1
25333-26259	308	CopB (Ps pv. syringae UMAF0081)	308/308(100%), 308/308(100%)	AFV33250.1
26256-28133	625	CopA (Ps pv. syringae UMAF0081)	625/625 (100%), 625/625 (100%)	AFV33251.1
28797-29192	131	Hp PsyrptA_21076 (Ps pv. tabaci ATCC 11528)	131/131 (100%), 131/131 (100%)	ZP_05639815.1
29394-29594	66	Twin-arginine translocation pathway signal:copper-resistance protein CopA (Ps pv. tabaci ATCC 11528)	66/66 (100%), 66/66 (100%)	ZP_05639814.1
30088-31050	320	Phage integrase:Phage integrase, N-terminal SAM-like protein (Ps pv. tabaci ATCC 11528)	320/320 (100%), 320/320 (100%)	ZP_05639813.1
31173-32288	371	Hp PsyrptA_21061 (Ps pv. tabaci ATCC 11528)	371/371 (100%), 371/371 (100%)	ZP_05639812.1
32340-33113	257	LuxR family transcriptional regulator (Ps pv. tabaci ATCC 11528)	257/257 (100%), 257/257 (100%)	ZP_05639811.1
33204-34322	372	ABC transporter (Ps pv. tomato NCPPB 1108)	372/372 (100%), 372/372 (100%)	ZP_07256281.1
34421-34618	65	Hp PSYTB_25019 (Ps pv. tabaci ATCC 11528)	65/65(100%), 65/65(100%)	gb|EGH92920.1
34630-35025	131	XRE/PbsX family transcriptional regulator (Ps pv. tabaci ATCC 11528)	131/131 (100%), 131/131 (100%)	ZP_05639808.1

35169-35753	194	Transcriptional regulator (plasmid)/termination factor nusG (*P. syringae* CC1557)	194/194 (100%), 194/194 (100%)	AHG43708.1
35842-36084	80	Hp PsyrptA_21031 (Ps pv. tabaci ATCC 11528)	80/80 (100%), 80/80 (100%)	ZP_05639806.1
36097-36801	234	VirB1 (Ps pv. tabaci ATCC 11528)	234/234 (100%), 234/234 (100%)	ZP_05639805.1
36801-37136	111	VirB2 (Ps pv. tabaci ATCC 11528)	111/111 (100%), 111/111 (100%)	ZP_05639804.1
37149-37637	162	VirB3 (Ps pv. tabaci ATCC 11528)	162/162(100%), 162/162(100%)	ZP_05639803.1
37564-40038	824	VirB4 (Ps pv. tabaci ATCC 11528)	824/824(100%), 824/824(100%)	ZP_05639802.1
40035-40715	226	VirB5 (Ps pv. tabaci ATCC 11528)	226/226 (100%), 226/226 (100%)	ZP_05639801.1
40731-40937	68	Hp PSYTB_24974 (Ps pv. tabaci str. ATCC 11528)	68/68(100%), 68/68(100%)	gb|EGH92911.1
40964-41908	314	VirB6 (Ps pv. tabaci ATCC 11528)	314/314 (100%), 314/314 (100%)	ZP_05639799.1
41969-42271	100	VirB7 (*P. syringae* BRIP39023)	95/100(95%), 97/100(97%)	ZP_20926108.1
42309-43097	262	VirB8 (Ps pv. tabaci ATCC 11528)	262/262 (100%), 262/262 (100%)	ZP_05639798.1
43087-43896	269	VirB9 (Ps pv. tabaci ATCC 11528)	269/269 (100%), 269/269 (100%)	ZP_05639797.1
43883-45253	456	VirB10 (Ps pv. tabaci ATCC 11528)	456/456 (100%), 456/456 (100%)	ZP_05639796.1
45263-46294	343	VirB11 (Ps pv. tabaci ATCC 11528)	343/343(100%), 343/343(100%)	ZP_05639795.1
46304-46660	118	Hp PsyrptN_02705 (Ps pv. tomato NCPPB 1108)	118/118 (100%), 118/118 (100%)	ZP_07256265.1
46780-47097	105	Hp PsyrptA_20966 (Ps pv. tabaci ATCC 11528)	105/105 (100%), 105/105 (100%)	ZP_05639793.1
47107-47340	68	Hp PSYTB_24974 (Ps pv. tabaci ATCC 11528)	68/68(100%), 68/68(100%)	gb|EGH92911.1
47340-48992	550	VirD4 (Ps pv. tomato NCPPB 1108)	550/550 (100%), 550/550 (100%)	ZP_07256262.1
49033-49395	120	Killer protein (Ps pv. tabaci ATCC 11528)	120/120 (100%), 120/120 (100%)	ZP_05639857.1
49423-51588	721	Orf50 (Ps pv. tabaci ATCC 11528)	721/721 (100%), 721/721 (100%)	ZP_05639856.1
51616-52383	255	Hp PsyrptA_21276 (Ps pv. tabaci ATCC 11528)	255/255 (100%), 255/255 (100%)	ZP_05639855.1
52448-53050	201	Single-strand DNA-binding protein (Ps pv. tabaci ATCC 11528)	201/201 (100%), 201/201 (100%)	ZP_05639854.1
53359-54093	244	Stability protein type I (Ps pv. tabaci ATCC 11528)	244/244 (100%), 244/244 (100%)	ZP_05639853.1
54090-54662	190	Hp PsyrptA_21261 (Ps pv. tabaci ATCC 11528)	190/190 (100%), 190/190 (100%)	ZP_05639852.1
54689-55228	179	Hp PsyrptA_21256 (Ps pv. tabaci ATCC 11528)	179/179 (100%), 179/179 (100%)	ZP_05639851.1
55453-56178	241	Orf53 (Ps pv. tabaci ATCC 11528)	240/241(99%), 241/241(100%)	ZP_05639850.1
56168-59917	1249	Orf54 (*P. syringae*)	1225/1249(98%), 1235/1249(98%)	YP_006963256.1
60047-60697	216	ParA family protein (Ps pv. tabaci ATCC 11528)	216/216 (100%), 216/216 (100%)	ZP_05639845.1
60688-61041	117	Hp PsyrptA_21221 (Ps pv. tabaci (ATCC 11528)	117/117 (100%), 117/117 (100%)	ZP_05639844.1

[a] P: *Pseudomonas*, Ps: *Pseudomonas syringae*, Hp: Hypothetical protein. [b] Gray boxes: Specific genes present in this plasmid (This plasmid sequence is identical at 99% to the plasmid of the strain Psy 6-9, harboring the same genes).

Table S3 Predicted ORFs in the *P. syringae* pv. syringae UMAF0170 plasmid

Position (5´-3´)	Size (aa)	Homology (Blast)[a]	Identity and similarity (source of homolog)	Accession No.
1-1314	437	RepA (Ps pv. tabaci ATCC 11528)	436/437 (99%), 436/437 (99%),	ZP_05639843.1
1453-1878	141	RulA (Ps pv. tabaci ATCC 11528)	141/141 (100%), 141/141 (100%),	ZP_05639842.1
1856-3154	432	RulB (Ps pv. syringae)	417/432 (97%), 426/432 (99%),	NP_940693.1
3230-3550	106	Orf8 / Hp (Ps pv. syringae)	106/106 (100%), 106/106 (100%),	NP_940694.1
3816-4103	95	Orf9 / Hp (Ps pv. syringae)	93/95 (98%), 94/95 (98%)	NP_940695.1
4191-4607	138	Orf10 / Hp (Ps pv. pisi 1704B)	138/138(100%), 138/138(100%)	ZP_16698741.1
4691-4852	53	Hp (*P. syringae*)	50/53(94%), 51/53(96%)	YP_006963270.1
4945-5145	66	Hp PsyrptA_21191 (Ps pv. tabaci ATCC 11528)	66/66 (100%), 66/66 (100%)	ZP_05639838.1
5258-5908	216	Plasmid partitioning protein ParA (*P. amygdali*)	214/216(99%), 216/216(100%)	WP_003348583.1
5898-6185	95	Orf12 / Hp (Ps pv. pisi 1704B)	92/95(97%), 95/95(100%)	ZP_16698744.1
6408-7049	213	Putative SOS response-associated peptidase YedK (*P. syringae*)	204/213(96%), 209/213(98%)	SDO16173.1
7987-8604[b]	205	Site-specific tyrosine recombinase / integrase (*P. syringae*)	199/205(97%), 202/205(98%)	WP_016569395.1
8939-9622	227	Hp (*P. syringae* group genomosp. 3)	188/227(83%), 206/227(90%)	WP_010204130.1
9722-10285	187	Metal binding protein (*P. syringae*)	187/187(100%), 187/187(100%)	YP_006963698.1
10356-11735	459	Heavy metal sensor kinase, CopS (Ps pv. japonica M301072)	458/459(99%), 459/459(100%)	ZP_16688559.1
11732-12418	228	Heavy metal response regulator, CopR (Ps pv. japónica M301072)	227/228(99%), 227/228(99%)	ZP_16688558.1
12474-13406	310	CopD (*P. syringae*)	310/310(100%), 310/310(100%)	YP_006963694.1
13409-13789	126	CopC (*P. syringae*)	126/126(100%), 126/126(100%)	YP_006963693.1
13852-14838	328	CopB (*P. syringae*)	328/328(100%), 328/328(100%)	YP_006963692.1
14844-16682	612	CopA (*P. syringae*)	609/612(99%), 609/612(99%)	gb|AAA25806.1
16811-17002	63	Hp (*P. syringae*)	61/63(97%), 63/63(100%)	WP_003317552.1
17132-18094	320	Phage integrase, N-terminal SAM-like (Ps pv. syringae B728a)	315/320 (98%), 316/320 (99%),	YP_234580.1
18370-19989	539	Histidine kinase, HAMP region: chemotaxis sensory transducer (Ps pv. syringae B728a)	539/539 (100%), 539/539 (100%)	YP_234579.1
20115-20492	125	Hp PSPTO_4623 (Ps pv. tomato DC3000)	125/125(100%), 125/125(100%)	NP_794374.1
20619-20897	92	Hp Psyr_1489 (Ps pv. syringae B728a)	91/92(99%), 92/92(100%)	YP_234577.1
20985-21224	79	Hp (Ps pv. actinidiae)	79/79(100%), 79/79(100%)	gb|AGE82257.1
21331-21765	144	MerR family transcriptional regulator (Ps pv. tomato DC3000)	143/144(99%), 143/144(99%)	NP_794373.1
21857-22600	247	arsenical-resistance protein ACR3 (Ps pv. actinidiae)	246/246(100%), 246/246(100%)	gb|ELQ02638.1
22611-22934	107	Hp PSPTO_4620 (Ps pv. tomato DC3000)	107/107(100%), 107/107(100%)	NP_794371.1
23183-24529	448	Cl- channel, voltage gated (Ps pv. syringae B728a)	448/448(100%), 448/448(100%)	YP_234574.1
24631-24762	43	Hp (Ps pv. actinidiae)	43/43(100%), 43/43(100%)	gb|AGE82623.1
24972-25295	107	Hp PSPTO_4618 (Ps pv. tomato DC3000)	107/107(100%), 107/107(100%)	NP_794369.1
25338-25604	88	Hp Psyr_1483 (Ps pv. syringae B728a)	88/88(100%), 88/88(100%)	YP_234571.1
25699-26040	113	Hp POR16_24233 (Ps pv. oryzae 1_6)	113/113(100%), 113/113(100%)	ZP_04590417.1
26114-27397	427	Phosphopyruvate hydratase (Ps pv. syringae B728a)	427/427 (100%), 427/427 (100%)	YP_234570.1
27513-28058	181	Inorganic pyrophosphatase (Ps pv. tomato DC3000)	181/181(100%), 181/181(100%)	NP_794366.1
28058-28660	200	IS1415 transposase istA/B / Uracil DNA glycosylase superfamily protein (*Pseudomonas*)	199/200(99%), 200/200(100%)	WP_003317532.1
28669-29838	389	C4-dicarboxylate transporter/malic acid transport protein (Ps pv. syringae B728a)	389/389 (100%), 389/389 (100%)	YP_234567.1
29956-30852	298	LysR family transcriptional regulator (Ps pv. tomato DC3000)	298/298(100%), 298/298(100%)	NP_794363.1

30962-32002	346	Surface antigen (Ps pv. actinidiae)	346/346(100%), 346/346(100%)	gb|AGE82241.1
32331-32711	126	Hp PSPTO_4610 (Ps pv. tomato DC3000)	126/126 (100%), 126/126 (100%)	NP_794361.1
33207-33452	81	Hp PSPTO_4609 (Ps pv. tomato DC3000)	80/81(99%), 81/81(100%)	NP_794360.1
33426-34388	320	Phage integrase:Phage integrase, N-terminal SAM-like (Ps pv. syringae B728a)	320/320 (100%), 320/320 (100%)	YP_234563.1
34511-35626	371	Hp PsyrptA_21061 (Ps pv. tabaci ATCC 11528)	368/371 (99%), 370/371 (99%)	ZP_05639812.1
35678-36451	257	LuxR family transcriptional regulator (Ps pv. tabaci ATCC 11528)	256/257 (99%), 256/257 (99%)	ZP_05639811.1
36542-37660	372	ABC transporter, periplasmic polyamine-binding protein (Ps pv. tomato NCPPB 1108)	370/372 (99%), 372/372 (100%)	ZP_07256281.1
37759-37956	65	Hp (*P. amygdali*)	65/65(100%), 65/65(100%)	WP_005782488.1
37968-38363	131	XRE/PbsX family transcriptional regulator (Ps pv. tabaci ATCC 11528)	128/131 (98%), 131/131 (100%)	ZP_05639808.1
38579-39112	177	Transcription antiterminator (plasmid) NusG (*P. syringae* pv. syringae HS191)	175/177(99%), 177/177(100%)	AKF48831.1
39201-39443	80	Hp PsyrptA_21031 (Ps pv. tabaci ATCC 11528)	78/80 (98%), 79/80 (99%)	ZP_05639806.1
39456-40160	234	VirB1 (Ps pv. tabaci ATCC 11528)	234/234 (100%), 234/234 (100%)	ZP_05639805.1
40160-40495	111	VirB2 (Ps pv. tabaci ATCC 11528)	111/111 (100%), 111/111 (100%),	ZP_05639804.1
40508-40996	162	VirB3 (Ps pv. tabaci ATCC 11528)	162/162 (100%), 162/162 (100%)	ZP_05639803.1
40845-43397	850	VirB4 (Ps pv. phaseolicola 1448A)	814/850 (96%), 838/850 (98%)	YP_272283.1
43394-44074	226	VirB5 (Ps pv. tabaci ATCC 11528)	226/226 (100%), 226/226 (100%)	ZP_05639801.1
44090-44296	68	Hp PsyrptA_21001 (Ps pv. tabaci ATCC 11528)	68/68 (100%), 68/68 (100%)	ZP_05639800.1
44323-45267	314	VirB6 (Ps pv. tabaci ATCC 11528)	310/314 (99%), 313/314 (99%)	ZP_05639799.1
45327-45611	94	VirB7 (*P. syringae* BRIP39023)	90/100(90%), 91/100(91%)	ZP_20926108.1
45649-46437	262	VirB8 (Ps pv. tabaci ATCC 11528)	262/262 (100%), 262/262 (100%)	ZP_05639798.1
46427-47236	269	VirB9 (Ps pv. tabaci ATCC 11528)	269/269 (100%), 269/269 (100%)	ZP_05639797.1
47223-48593	456	VirB10 (Ps pv. tabaci ATCC 11528)	453/456 (99%), 454/456 (99%)	ZP_05639796.1
48603-49634	343	VirB11 (Ps pv. tabaci ATCC 11528)	343/343(100%), 343/343(100%)	ZP_05639795.1
49644-50000	118	Hp (*P. syringae* group genomosp. 3)	118/118(100%), 118/118(100%)	WP_010217013.1
50284-51945	553	VirD4 (*P. syringae*)	543/553(98%), 548/553(99%)	YP_006963352.1
51986-52348	120	Killer protein (Ps pv. tabaci ATCC 11528)	118/120 (98%), 119/120 (99%)	ZP_05639857.1
52376-54541	721	Orf50 (Ps pv. tabaci ATCC 11528)	720/721 (99%), 721/721 (100%)	ZP_05639856.1
54569-55336	255	Hp PsyrptA_21276 (Ps pv. tabaci ATCC 11528)	254/255 (99%), 254/255 (99%)	ZP_05639855.1
55401-56006	201	Single-stranded DNA-binding protein (Ps pv. tabaci ATCC 11528)	201/201 (100%), 201/201 (100%)	ZP_05639854.1
56312-57046	244	Stability protein type I (Ps pv. tabaci ATCC 11528)	243/248 (98%), 245/248 (100%)	NP_940738.1
57043-57615	190	Hp PsyrptA_21261 (Ps pv. tabaci ATCC 11528)	190/190 (100%), 190/190 (100%)	ZP_05639852.1
57642-58181	179	Hp PsyrptA_21256 (Ps pv. tabaci ATCC 11528)	178/179 (99%), 179/179 (100%)	ZP_05639851.1
58385-59131	241	Orf53 (Ps pv. syringae)	239/241(99%), 240/241(99%)	ZP_05639850.1
59121-62870	1249	Orf54 (*P. syringae*)	1225/1249(98%), 1233/1249(98%)	YP_006963256.1
63000-63650	216	ParA (Ps pv. tabaci ATCC 11528)	216/216(100%), 216/216(100%)	ZP_05639845.1
63641-63994	117	Hp PsyrptA_21221 (Ps pv. tabaci ATCC 11528)	117/117 (100%), 117/117 (100%)	ZP_05639844.1

[a] P: *Pseudomonas*, Ps: *Pseudomonas syringae*, Hp: Hypothetical protein. [b] Gray boxes: Specific genes present in this plasmid.

Table S4 Predicted ORFs in the *P. syringae* pv. syringae UMAF0158 plasmid

Position (5´-3´)	Size (aa)	Homology (Blast)[a]	Identity and similarity (source of homolog)	Accesion No.
1-1302	433	RepA (Ps pv. lachrymans M302278)	407/437(93%), 419/437(95%)	WP_005769737.1
1364-2071	235	Hp (*P. stutzeri*)	177/235(75%), 203/235(86%)	WP_021206349.1
2430-2717	95	Hp (*P. syringae*)	58/91(64%), 73/91(80%)	WP_020303940.1
2986-3942	318	6-hydroxy-3-succinoylpyridine hydroxylase NicB (P. stutzeri)	279/318(88%), 298/318(93%)	WP_021206355.1
4143-4568	141	RulA (Ps pv. tomato str. DC3000)	139/141 (99%), 140/141 (99%)	NP_808664.1
4546-5844	432	RulB (Ps pv. syringae)	413/432(96%), 420/432(97%)	WP_017279392.1
6006-6293	95	Hp ALO82_200232 (Ps pv. broussonetiae)	95/95(100%), 95/95(100%)	KPW64255.1
6383-6790	135	Hp (*P. syringae* group)	135/135 (100%), 135/135 (100%)	WP_060709006.1
6874-7035	53	Hp (*P. syringae*)	49/53(92%), 50/53(94%)	YP_006963270.1
7128-7328	66	Hp (*P. syringae* group)	62/66 (94%), 62/66 (94%)	WP_003348581.1
7442-8092	216	ParA (Ps pv. maculicola str. M6)	213/216(99%), 215/216(99%)	NP_114201.1
8082-8369	95	Hp (*P. syringae*) (Orf12)	95/95(100%), 95/95(100%)	WP_024639839.1
8777-9316	179	Hp (*P. amygdali*)	174/179(97%), 177/179(98%)	WP_005735278.1
9592-10233	213	Hp (*P. syringae* group)	202/213(95%), 209/213(98%)	WP_003436862.1
10621-11967[b]	448	MFS transporter (*P. syringae*)	448/448(100%), 448/448(100%)	WP_060709012.1
12034-12933	299	LysR TR (*P. rhizosphaerae*)	250/298(84%), 280/298(93%)	WP_043193607.1
13033-13659	208	Methyltransferase / ribonuclease (*P. syringae*)	208/208(100%), 208/208(100%)	WP_060709013.1
13676-14545	289	3-hydroxyisobutyrate dehydrogenase (*P. abietaniphila*)	264/289(91%), 277/289(95%)	SDH93023.1
15570-16268	232	Haloacid dehalogenase	175/232(75%), 196/232(84%)	WP_037013522.1
16851-17174	107	Negative regulator GrlR (*P. syringae* BRIP39023)	107/107(100%), 107/107(100%)	ZP_20925266.1
17612-17863	83	Antirestriction protein ArdR (*P. syringae*)	80/83(96%), 81/83(97%)	WP_024690828.1
18758-21403	881	chromosome segregation protein SMC (*P. syringae*)	881/881(100%), 881/881(100%)	WP_060709021.1
21437-21685	82	Hp (*P. syringae*)	78/81(96%), 79/81(97%)	WP_003433292.1
21758-22036	92	Hp (*P. syringae* group)	90/92(98%), 91/92(98%)	WP_003436873.1
22054-24090	678	Methyl-accepting chemotaxis protein (*P. syringae* group)	670/678(99%), 674/678(99%)	WP_003436874.1
25876-28689	937	PAS/PAC sensor hybrid histidine kinase (Ps pv. morsprunorum M302280)	641/921(70%), 746/921(80%)	ZP_16666750.1
28888-29298	136	IS3 family transposase (*P. putida* DOT-T1E)	75/86(87%), 82/86(95%)	YP_006533700.1
29453-29968	171	Diguanylate cyclase (*P. amygdal)*	157/171(92%), 164/171(95%)	WP_005740768.1
30171-31016	281	Regulatory protein, LuxR (Ps pv. syringae FF5)	223/276(81%), 244/276(88%)	EGH79899.1
31306-32265	319	Phage integrase family (*P. syringae* BRIP39023)	304/319(95%), 307/319(96%)	ZP_20925307.1
32483-32983	166	Hcp1 effector type VI (*P. syringae*)	165/166(99%), 165/166(99%)	WP_016569397.1
33008-33535	175	Hp (Ps pv. syringae SM)	173/175(99%), 174/175(99%)	EPF64134.1
33900-34673	257	LuxR TR (*P. syringae*)	256/257(99%), 256/257(99%)	WP_016569400.1
34764-35882	372	ABC transporter (Ps pv. tabaci str. ATCC 11528)	330/372(89%), 358/372(96%)	EGH92921.1
35979-36194	71	Hp (*P. syringae*)	69/71(97%), 69/71(97%)	WP_016569402.1
36204-36548	114	XRE family transcriptional regulator (P. syringae)	113/114(99%), 114/114(100%)	WP_032635022.1
36795-37328	177	Transcriptional regulator (plasmid) (*P. syringae* UMAF0158) (NusG)	176/177(99%), 177/177(100%)	ALE01072.1
37417-37659	80	Hp (*P. syringae*)	78/80(98%), 79/80(98%)	WP_015061857.1
37672-38379	235	VirB1 (P. syringae)	228/235(97%), 229/235(97%)	WP_016569405.1
38376-38714	112	VirB2 (Ps pv. glycinea race 4)	104/112(93%), 108/112(96%)	EGH17791.1
38727-39215	162	VirB3 (*P. syringae*)	159/162(98%), 162/162(100%)	YP_006963339.1

39064-41616	850	VirB4 (s pv. tabaci str. ATCC 11528)	815/850(96%), 836/850(98%)	KEZ65865.1
41613-42299	228	VirB5 (*P. syringae*)	217/228(95%), 222/228(97%)	WP_016569409.1
42332-42685	117	Hp (*P. syringae*)	108/117(92%), 115/117(98%)	WP_024668748.1
42696-43628	310	VirB6 (Ps pv. aceris M302273)	294/310(95%), 303/310(97%)	ZP_16735656.1
43696-43998	100	VirB7 (*P. syringae*)	93/100(93%), 93/100(93%)	WP_003436825.1
44036-44824	262	VirB8 (*P. syringae*)	260/262(99%), 262/262(100%)	YP_006963345.1
44814-45623	269	VirB9 (*P. syringae*)	263/269(98%), 265/269(98%)	YP_006963346.1
45610-46980	456	VirB10 (*P. syringae*)	430/456(94%), 445/456(97%)	YP_006963347.1
46990-48021	343	VirB11 (Ps pv. syringae)	337/343(98%), 339/343(98%)	WP_003436829.1
48031-48387	118	Hp (*P. syringae*)	116/118(98%), 118/118(100%)	YP_006963349.1
48514-48744	76	Hp (*P. syringae*]	75/76(99%), 75/76(98%)	WP_016569418.1
48744-50396	550	VirD4 (*P. syringae*)	515/550(94%), 536/550(97%)	YP_006963352.1
50438-50800	120	Killer protein (*P. syringae*)	115/120(96%), 117/120(97%)	WP_025391089.1
50828-52993	721	Orf50 (*P. syringae* group)	710/721 (98%), 715/721 (99%)	WP_003436835.1
53021-53785	254	Hp (*P. syringae* group)	245/255(96%), 250/255(98%)	WP_010199959.1
53850-54455	201	single-stranded DNA-binding protein (*P. syringae*)	193/201(96%), 195/201(97%)	WP_024639857.1
54762-55505	247	Stability protein (Ps pv. syringae)	238/247 (96%), 243/247 (98%)	NP_940737.1
55502-56074	190	Hp (*P. syringae*)	186/190(98%), 187/190(98%)	WP_003407072.1
56101-56523	140	Hp (*P. syringae*)	131/140(94%), 138/140(98%)	WP_032618937.1
56844-57590	248	Orf53 (*P. syringae*)	241/248(97%), 242/248(97%)	WP_011152919.1
57580-61323	1247	Orf54 (Ps pv. syringae)	1176/1251 (94%), 1212/1251 (96%)	NP_940739.1
61453-62103	216	ParA family protein (*P. syringae* group)	216/216 (100%), 216/216 (100%)	WP_003407061.1
62094-62447	117	Hp (*P. syringae*)	116/117(99%), 116/117(99%)	WP_003436847.1
62490-62663	57	Hp (*P. syringae*)	45/54(83%), 49/54(90%)	YP_006963259.1

[a] P: *Pseudomonas*, Ps: *Pseudomonas syringae*, Hp: Hypothetical protein. [b] Gray boxes: Specific genes present in this plasmid (This plasmid sequence varies from the plasmid sequence of the strain Psy UMAF1029 just in 56 bp, not altering the genes present in both plasmids).

Table S5 Predicted ORFs in the *P. syringae* pv. syringae UMAF1029 plasmid

**Position (5´-3´)**	**Size (aa)**	**Homology (Blast)**	**Identity and similarity (source of homolog)**	**Accession No.**
1-1302	433	RepA (Ps pv. lachrymans M302278)	407/437(93%), 419/437(95%)	WP_005769737.1
1364-2071	235	Hp (*P. stutzeri*)	177/235(75%), 203/235(86%)	WP_021206349.1
2430-2717	95	Hp (*P. syringae*)	58/91(64%), 73/91(80%)	WP_020303940.1
2986-3942	318	6-hydroxy-3-succinoylpyridine hydroxylase NicB (P. stutzeri)	279/318(88%), 298/318(93%)	WP_021206355.1
4143-4568	141	RulA (Ps pv. tomato str. DC3000)	139/141 (99%), 140/141 (99%)	NP_808664.1
4546-5844	432	RulB (Ps pv. syringae)	413/432(96%), 420/432(97%)	WP_017279392.1
6006-6293	95	Hp ALO82_200232 (Ps pv. broussonetiae)	95/95(100%), 95/95(100%)	KPW64255.1
6390-6797	135	Hp (P. syringae group)	135/135 (100%), 135/135 (100%)	WP_060709006.1
6881-7042	53	Hp (*P. syringae*)	49/53(92%), 50/53(94%)	YP_006963270.1
7135-7335	66	Hp (*P. syringae* group)	62/66 (94%), 62/66 (94%)	WP_003348581.1
7449-8099	216	ParA (Ps pv. maculicola str. M6)	213/216(99%), 215/216(99%)	NP_114201.1
8089-8376	95	Hp (*P. syringae*) (Orf12)	95/95(100%), 95/95(100%)	WP_024639839.1
8784-9323	179	Hp (*P. amygdali*)	174/179(97%), 177/179(98%)	WP_005735278.1
9599-10240	213	Hp (*P. syringae* group)	202/213(95%), 209/213(98%)	WP_003436862.1
10628-11974[b]	448	MFS transporter (*P. syringae*)	448/448(100%), 448/448(100%)	WP_060709012.1
12041-12940	299	LysR TR (*P. rhizosphaerae*)	250/298(84%), 280/298(93%)	WP_043193607.1
13040-13666	208	Methyltransferase / ribonuclease (*P. syringae*)	208/208(100%), 208/208(100%)	WP_060709013.1
13683-14552	289	3-hydroxyisobutyrate dehydrogenase (*P. abietaniphila*)	264/289(91%), 277/289(95%)	SDH93023.1
15577-16275	232	Haloacid dehalogenase	175/232(75%), 196/232(84%)	WP_037013522.1
16858-17181	107	Negative regulator GrlR (*P. syringae* BRIP39023)	107/107(100%), 107/107(100%)	ZP_20925266.1
17619-17870	83	Antirestriction protein ArdR (*P. syringae*)	80/83(96%), 81/83(97%)	WP_024690828.1
18765-21410	881	chromosome segregation protein SMC (*P. syringae*)	881/881(100%), 881/881(100%)	WP_060709021.1
21444-21692	82	Hp (*P. syringae*)	78/81(96%), 79/81(97%)	WP_003433292.1
21765-22043	92	Hp (*P. syringae* group)	90/92(98%), 91/92(98%)	WP_003436873.1
22061-24097	678	Methyl-accepting chemotaxis protein (*P. syringae* group)	670/678(99%), 674/678(99%)	WP_003436874.1
25883-28696	937	PAS/PAC sensor hybrid histidine kinase (Ps pv. morsprunorum M302280)	641/921(70%), 746/921(80%)	ZP_16666750.1
28895-29305	136	IS3 family transposase (*P. putida* DOT-T1E)	75/86(87%), 82/86(95%)	YP_006533700.1
29460-29975	171	Diguanylate cyclase (*P. amygdal)*	157/171(92%), 164/171(95%)	WP_005740768.1
30178-31023	281	Regulatory protein, LuxR (Ps pv. syringae FF5)	223/276(81%), 244/276(88%)	EGH79899.1
31313-32272	319	Phage integrase family (*P. syringae* BRIP39023)	304/319(95%), 307/319(96%)	ZP_20925307.1
32490-32990	166	Hcp1 effector type VI (*P. syringae*)	165/166(99%), 165/166(99%)	WP_016569397.1
33015-33542	175	Hp (Ps pv. syringae SM)	173/175(99%), 174/175(99%)	EPF64134.1
33906-34679	257	LuxR TR (*P. syringae*)	256/257(99%), 256/257(99%)	WP_016569400.1
34770-35888	372	ABC transporter (Ps pv. tabaci str. ATCC 11528)	330/372(89%), 358/372(96%)	EGH92921.1
35985-36200	71	Hp (*P. syringae*)	69/71(97%), 69/71(97%)	WP_016569402.1
36210-36554	114	XRE family transcriptional regulator (P. syringae)	113/114(99%), 114/114(100%)	WP_032635022.1
36801-37334	177	Transcriptional regulator (plasmid) (*P. syringae* UMAF0158) (NusG)	176/177(99%), 177/177(100%)	ALE01072.1
37423-37665	80	Hp (*P. syringae*)	78/80(98%), 79/80(98%)	WP_015061857.1
37678-38385	235	VirB1 (P. syringae)	228/235(97%), 229/235(97%)	WP_016569405.1
38382-38720	112	VirB2 (Ps pv. glycinea race 4)	104/112(93%), 108/112(96%)	EGH17791.1
38733-39221	162	VirB3 (*P. syringae*)	159/162(98%), 162/162(100%)	YP_006963339.1
39070-41622	850	VirB4 (s pv. tabaci str. ATCC 11528)	815/850(96%), 836/850(98%)	KEZ65865.1

41619-42305	228	VirB5 (*P. syringae*)	217/228(95%), 222/228(97%)	WP_016569409.1
42338-42691	117	Hp (*P. syringae*)	108/117(92%), 115/117(98%)	WP_024668748.1
42702-43634	310	VirB6 (Ps pv. aceris M302273)	294/310(95%), 303/310(97%)	ZP_16735656.1
43702-44004	100	VirB7 (*P. syringae*)	93/100(93%), 93/100(93%)	WP_003436825.1
44042-44830	262	VirB8 (*P. syringae*)	260/262(99%), 262/262(100%)	YP_006963345.1
44820-45629	269	VirB9 (*P. syringae*)	263/269(98%), 265/269(98%)	YP_006963346.1
45616-46986	456	VirB10 (*P. syringae*)	430/456(94%), 445/456(97%)	YP_006963347.1
46996-48027	343	VirB11 (Ps pv. syringae)	337/343(98%), 339/343(98%)	WP_003436829.1
48037-48393	118	Hp (*P. syringae*)	116/118(98%), 118/118(100%)	YP_006963349.1
48520-48750	76	Hp (*P. syringae*]	75/76(99%), 75/76(98%)	WP_016569418.1
48750-50402	550	VirD4 (*P. syringae*)	515/550(94%), 536/550(97%)	YP_006963352.1
50444-50806	120	Killer protein (*P. syringae*)	115/120(96%), 117/120(97%)	WP_025391089.1
50834-52999	721	Orf50 (*P. syringae* group)	710/721 (98%), 715/721 (99%)	WP_003436835.1
53027-53791	254	Hp (*P. syringae* group)	245/255(96%), 250/255(98%)	WP_010199959.1
53856-54461	201	single-stranded DNA-binding protein (*P. syringae*)	193/201(96%), 195/201(97%)	WP_024639857.1
54768-55511	247	Stability protein (Ps pv. syringae)	238/247 (96%), 243/247 (98%)	NP_940737.1
55508-56080	190	Hp (*P. syringae*)	186/190(98%), 187/190(98%)	WP_003407072.1
56107-56529	140	Hp (*P. syringae*)	131/140(94%), 138/140(98%)	WP_032618937.1
56871-57596	241	Orf53 (*P. syringae*)	241/248(97%), 242/248(97%)	WP_011152919.1
57586-61332	1248	Orf54 (Ps pv. syringae)	1176/1251 (94%), 1212/1251 (96%)	NP_940739.1
61462-62112	216	ParA family protein (*P. syringae* group)	216/216 (100%), 216/216 (100%)	WP_003407061.1
62103-62456	117	Hp (*P. syringae*)	116/117(99%), 116/117(99%)	WP_003436847.1
62499-62663	54	Hp (*P. syringae*)	45/54(83%), 49/54(90%)	YP_006963259.1

[a] P: *Pseudomonas*, Ps: *Pseudomonas syringae*, Hp: Hypothetical protein. [b] Gray boxes: Specific genes present in this plasmid (This plasmid sequence varies from the plasmid sequence of the strain Psy UMAF0158 just in 56 bp, not altering the genes present in both plasmids).

Table S6 Predicted ORFs in the *P. syringae* pv. syringae 6-9 plasmid

Position (5´-3´)	Size (aa)	Homology (Blast)[a]	Identity and similarity (source of homolog)	Accession No.
1-1314	437	RepA (Ps pv. tabaci ATCC 11528)	437/437 (100%), 437/437 (100%),	ZP_05639843.1
1453-1878	141	RulA (Ps pv. tabaci ATCC 11528)	141/141 (100%), 141/141 (100%)	ZP_05639842.1
1856-3154	432	RulB (Ps pv. tabaci ATCC 11528)	426/427(99%), 427/427(100%)	ZP_05639841.1
3230-3535	101	Hp (*P. syringae* group)	101/101(100%), 101/101(100%)	WP_005782530.1
3816-4103	95	Hp ALO82_200232 (Ps pv. broussonetiae)	94/95(99%), 95/95(100%)	KPW64255.1
4197-4607	136	Orf10/Hp (Ps pv. tabaci ATCC 11528)	136/136 (100%), 136/136 (100%)	WP_005782529.1
4691-4852	53	Hp (*P. syringae*)	50/53(94%), 51/53(96%)	YP_006963270.1
4945-5145	66	Hp PsyrptA_21191 (Ps pv. tabaci ATCC 11528)	66/66 (100%), 66/66 (100%)	ZP_05639838.1
5258-5908	216	Plasmid partitioning protein ParA (*P. amygdali*)	216/216(100%), 216/216(100%)	WP_003348583.1
5898-6185	95	Orf12/Hp (Ps pv. tabaci ATCC 11528)	95/95 (100%), 95/95 (100%)	ZP_05639836.1
6822-7211	129	Hp (*P. syringae* group)	129/129 (100%), 129/129 (100%)	WP_010199903.1
7390-8031	213	Hp PsyrptN_02905 (Ps pv. tomato NCPPB 1108)	213/213(100%), 213/213(100%)	ZP_07256350.1
8321-9007	228	Hp PsyrptA_21166 (Ps pv. tabaci ATCC 11528)	228/228 (100%), 228/228 (100%)	ZP_05639833.1
9023-9553	176	Hp PsyrptA_21161 (Ps pv. tabaci ATCC 11528)	176/176 (100%), 176/176 (100%)	ZP_05639832.1
9658-10617	319	Orf28/integrase (Ps pv. tabaci ATCC 11528)	319/319 (100%), 319/319 (100%)	ZP_05639831.1
10681-11361	226	Hp PsyrptA_21151/metal-binding protein (Ps pv. tabaci ATCC 11528)	226/226 (100%), 226/226 (100%)	ZP_05639830.1
11431-12810	459	Heavy metal sensor kinase, CopS (Ps pv. syringae UMAF0081)	459/459 (100%), 459/459 (100%)	AFV33241.1
12807-13493	228	Heavy metal response regulator, CopR (Ps pv. syringae UMAF0081)	228/228 (100%), 228/228 (100%)	AFV33242.1
13724-13921[b]	65	Heavy metal transport/detoxification protein/copper chaperone/CopZ (Ps pv. tabaci ATCC 11528)	65/65(100%), 65/65(100%)	gb|EGH92938.1
14079-16463	794	CopG (Ps pv. syringae UMAF0081)	794/794 (100%), 794/794 (100%)	AFV33243.1
16460-16858	132	Transcriptional regulator (*P. amygdali*) (Cu-(I) responsive trans. regulator	131/132(99%), 132/132(100%)	WP_005782518.1
16946-20092	1048	CusA (Ps pv. syringae UMAF0081)	1048/1048(100%), 1048/1048 (100%)	AFV33244.1
20089-21543	484	CusB (Ps pv. syringae UMAF0081)	484/484 (100%), 484/484 (100%)	AFV33245.1
21540-22802	420	CusC (Ps pv. syringae UMAF0081)	420/420 (100%), 420/420 (100%)	AFV33246.1
23416-23862	148	metal-binding protein (*P. amygdali* pv. tabaci str. ATCC 11528]	148/148 (100%), 148/148 (100%)	AFV33258.1
23951-24952	333	CopD (Ps pv. syringae UMAF0081)	333/333(100%), 333/333(100%)	AFV33248.1
24889-25272	127	CopC (Ps pv. syringae UMAF0081)	127/127 (100%), 127/127 (100%)	AFV33249.1
25333-26259	308	CopB (Ps pv. syringae UMAF0081)	308/308(100%), 308/308(100%)	AFV33250.1
26256-28133	625	CopA (Ps pv. syringae UMAF0081)	625/625 (100%), 625/625 (100%)	AFV33251.1
28797-29192	131	Hp PsyrptA_21076 (Ps pv. tabaci ATCC 11528)	131/131 (100%), 131/131 (100%)	ZP_05639815.1
29394-29594	66	Twin-arginine translocation pathway signal:copper-resistance protein CopA (Ps pv. tabaci ATCC 11528)	66/66 (100%), 66/66 (100%)	ZP_05639814.1
30088-31050	320	Phage integrase:Phage integrase, N-terminal SAM-like protein (Ps pv. tabaci ATCC 11528)	320/320 (100%), 320/320 (100%)	ZP_05639813.1
31173-32288	371	Hp PsyrptA_21061 (Ps pv. tabaci ATCC 11528)	371/371 (100%), 371/371 (100%)	ZP_05639812.1
32340-33113	257	LuxR family transcriptional regulator (Ps pv. tabaci ATCC 11528)	257/257 (100%), 257/257 (100%)	ZP_05639811.1
33204-34322	372	ABC transporter (Ps pv. tomato NCPPB 1108)	372/372 (100%), 372/372 (100%)	ZP_07256281.1
34421-34618	65	Hp PSYTB_25019 (Ps pv. tabaci ATCC 11528)	65/65(100%), 65/65(100%)	gb|EGH92920.1
34630-35025	131	XRE/PbsX family transcriptional regulator (Ps pv. tabaci ATCC 11528)	131/131 (100%), 131/131 (100%)	ZP_05639808.1

35169-35753	194	Transcriptional regulator (plasmid)/termination factor nusG (*P. syringae* CC1557)	194/194 (100%), 194/194 (100%)	AHG43708.1
35842-36084	80	Hp PsyrptA_21031 (Ps pv. tabaci ATCC 11528)	80/80 (100%), 80/80 (100%)	ZP_05639806.1
36097-36801	234	VirB1 (Ps pv. tabaci ATCC 11528)	234/234 (100%), 234/234 (100%)	ZP_05639805.1
36801-37136	111	VirB2 (Ps pv. tabaci ATCC 11528)	111/111 (100%), 111/111 (100%)	ZP_05639804.1
37149-37637	162	VirB3 (Ps pv. tabaci ATCC 11528)	162/162(100%), 162/162(100%)	ZP_05639803.1
37564-40038	824	VirB4 (Ps pv. tabaci ATCC 11528)	824/824(100%), 824/824(100%)	ZP_05639802.1
40035-40715	226	VirB5 (Ps pv. tabaci ATCC 11528)	226/226 (100%), 226/226 (100%)	ZP_05639801.1
40731-40937	68	Hp PSYTB_24974 (Ps pv. tabaci str. ATCC 11528)	68/68(100%), 68/68(100%)	gb|EGH92911.1
40964-41908	314	VirB6 (Ps pv. tabaci ATCC 11528)	314/314 (100%), 314/314 (100%)	ZP_05639799.1
41969-42271	100	VirB7 (*P. syringae* BRIP39023)	95/100(95%), 97/100(97%)	ZP_20926108.1
42309-43097	262	VirB8 (Ps pv. tabaci ATCC 11528)	262/262 (100%), 262/262 (100%)	ZP_05639798.1
43087-43896	269	VirB9 (Ps pv. tabaci ATCC 11528)	269/269 (100%), 269/269 (100%)	ZP_05639797.1
43883-45253	456	VirB10 (Ps pv. tabaci ATCC 11528)	456/456 (100%), 456/456 (100%)	ZP_05639796.1
45263-46294	343	VirB11 (Ps pv. tabaci ATCC 11528)	343/343(100%), 343/343(100%)	ZP_05639795.1
46304-46660	118	Hp PsyrptN_02705 (Ps pv. tomato NCPPB 1108)	118/118 (100%), 118/118 (100%)	ZP_07256265.1
46780-47097	105	Hp PsyrptA_20966 (Ps pv. tabaci ATCC 11528)	105/105 (100%), 105/105 (100%)	ZP_05639793.1
47107-47340	68	Hp PSYTB_24974 (Ps pv. tabaci ATCC 11528)	68/68(100%), 68/68(100%)	gb|EGH92911.1
47340-48992	550	VirD4 (Ps pv. tomato NCPPB 1108)	550/550 (100%), 550/550 (100%)	ZP_07256262.1
49033-49395	120	Killer protein (Ps pv. tabaci ATCC 11528)	120/120 (100%), 120/120 (100%)	ZP_05639857.1
49423-51588	721	Orf50 (Ps pv. tabaci ATCC 11528)	721/721 (100%), 721/721 (100%)	ZP_05639856.1
51616-52383	255	Hp PsyrptA_21276 (Ps pv. tabaci ATCC 11528)	255/255 (100%), 255/255 (100%)	ZP_05639855.1
52448-53050	201	Single-strand DNA-binding protein (Ps pv. tabaci ATCC 11528)	201/201 (100%), 201/201 (100%)	ZP_05639854.1
53359-54093	244	Stability protein type I (Ps pv. tabaci ATCC 11528)	244/244 (100%), 244/244 (100%)	ZP_05639853.1
54090-54662	190	Hp PsyrptA_21261 (Ps pv. tabaci ATCC 11528)	190/190 (100%), 190/190 (100%)	ZP_05639852.1
54689-55228	179	Hp PsyrptA_21256 (Ps pv. tabaci ATCC 11528)	179/179 (100%), 179/179 (100%)	ZP_05639851.1
55453-56178	241	Orf53 (Ps pv. tabaci ATCC 11528)	240/241(99%), 241/241(100%)	ZP_05639850.1
56168-59917	1249	Orf54 (*P. syringae*)	1225/1249(98%), 1235/1249(98%)	YP_006963256.1
60047-60697	216	ParA family protein (Ps pv. tabaci ATCC 11528)	216/216 (100%), 216/216 (100%)	ZP_05639845.1
60688-61041	117	Hp PsyrptA_21221 (Ps pv. tabaci (ATCC 11528)	117/117 (100%), 117/117 (100%)	ZP_05639844.1

[a] P: *Pseudomonas*, Ps: *Pseudomonas syringae*, Hp: Hypothetical protein. [b] Gray boxes: Specific genes present in this plasmid (This plasmid sequence is identical at 99% to the plasmid of the strain Psy UMAF0081, harboring the same genes).

Table S7 Predicted ORFs in the *P. syringae* pv. syringae 7B44 plasmid

Position (5´-3´)	Size (aa)	Homology (Blast)[a]	Identity and similarity (source of homolog)	Accession No.
1-1314	437	RepA (Ps pv. syringae)	436/437(99%), 436/437(99%)	gb|AAW01458.1
1453-1878	141	RulA (Ps pv. syringae)	141/141(100%), 141/141(100%)	gb|AAG39349.1
1856-3254	432	RulB (Ps pv. syringae)	422/432(98%), 426/432(98%)	NP_940693.1
3315-3602	95	Orf9 / Hp (Ps pv. syringae)	94/95(99%), 94/95(99%)	NP_940695.1
3690-4106	138	Orf10 / Hp (Ps pv. pisi 1704B)	138/138(100%), 138/138(100%)	ZP_16698741.1
4190-4351	53	Hp (*P. syringae*)	50/53(94%), 51/53(96%)	YP_006963270.1
4444-4644	66	Hp PsyrptA_21191 (Ps pv. tabaci ATCC 11528)	66/66(100%), 66/66(100%)	ZP_05639838.1
4757-5407	216	Plasmid partitioning protein ParA (*P. amygdali*)	216/216(100%), 216/216(100%)	WP_003348583.1
5397-5684	95	Orf12 / Hp (Ps pv. pisi 1704B)	95/95(100%), 95/95(100%)	ZP_16698744.1
5896-6537	213	Putative SOS response-associated peptidase YedK (*Pseudomonas* sp. BS3759	210/213(99%), 211/213(99%)	SDO75453.1
6725-7300	191	Hp PSYPI_29394 (Ps pv. pisi 1704B)	189/191(99%), 190/191(99%)	ZP_16698746.1
8287-9210	307	Integrase (Ps pv. syringae)	239/281(85%), 257/281(91%)	NP_940713.1
9934-10887[b]	317	Cation efflux protein (Ps pv. syringae B728a)	316/317(99%), 316/317(99%)	YP_234594.1
10887-11162	91	DNA-binding transcriptional regulator, FrmR family (*P. syringae*)	91/91(100%), 91/91(100%)	WP_011267062.1
11314-11715	133	Regulatory protein ArsR (Ps pv. syringae B728a)	133/133(100%), 133/133(100%)	YP_234592.1
11737-13026	429	Arsenical pump membrane protein ArsB (Ps pv. syringae B728a)	429/429(100%), 429/429(100%)	YP_234591.1
13035-13505	156	ArsC family transcriptional regulator (*P. syringae*)	156/156(100%), 156/156(100%)	WP_011267059.1
13505-14239	244	NADPH-dependent FMN reductase, ArsH (Ps pv. syringae B728a)	244/244(100%), 244/244(100%)	YP_234589.1
14269-14823	184	N-acetyltransferase GCN5 (Ps pv. syringae B728a)	184/184(100%), 184/184(100%)	YP_234588.1
14859-15320	153	Metal binding protein (*P. syringae*)	150/153(98%), 151/153(98%)	YP_006963698.1
15390-16769	459	Heavy metal sensor kinase, CopS (Ps pv. syringae B728a)	458/459(99%), 459/459(100%)	YP_234586.1
16766-17452	228	Heavy metal response regulator, CopR (Ps pv. syringae B728a)	228/228(100%), 228/228(100%)	YP_234585.1
17508-18440	310	CopD (Ps pv. syringae B728a)	310/310(100%), 310/310(100%)	YP_234584.1
18443-18823	126	CopC (Ps pv. syringae B728a)	126/126(100%), 126/126(100%)	YP_234583.1
18888-19826	312	CopB (Ps pv. syringae B728a)	312/312(100%), 312/312(100%)	YP_234582.1
19832-21652	606	CopA (Ps pv. syringae B728a)	606/606(100%), 606/606(100%)	YP_234581.1
21781-21987	68	Hp (*P. syringae*)	59/68(87%), 61/68(89%)	WP_003317552.1
22119-23081	320	Phage integrase:Phage integrase, N-terminal SAM-like (Ps pv. syringae B728a)	320/320 (100%), 320/320 (100%)	YP_234580.1
23357-24976	539	Histidine kinase, HAMP region: chemotaxis sensory transducer (Ps pv. syringae B728a)	539/539(100%), 539/539(100%)	YP_234579.1
25102-25479	125	Hp Psyr_1490 (Ps pv. syringae B728a)	125/125(100%), 125/125(100%)	YP_234578.1
25606-25884	92	Hp Psyr_1489 (Ps pv. syringae B728a)	91/92(99%), 92/92(100%)	YP_234577.1
26318-26752	144	MerR family transcriptional regulator (Ps pv. tomato DC3000)	144/144(100%), 144/144(100%)	NP_794373.1
26844-27587	247	arsenical-resistance protein ACR3 (Ps pv. actinidiae)	246/246(100%), 246/246(100%)	gb|ELQ02638.1
27598-27921	107	Hp Psyr_1487 (Ps pv. syringae B728a)	107/107(100%), 107/107(100%)	YP_234575.1
28158-29516	452	Voltage-gated chloride channel family protein (Ps pv. tomato DC3000)	451/452(99%), 452/452(100%)	NP_794370.1
29513-29878	121	Hp Psyr_1485 (Ps pv. syringae B728a)	121/121(100%), 121/121(100%)	YP_234573.1
29959-30282	107	Hp Psyr_1484 (Ps pv. syringae B728a)	107/107(100%), 107/107(100%)	YP_234572.1
30325-30591	88	Hp Psyr_1483 (Ps pv. syringae B728a)	88/88(100%), 88/88(100%)	YP_234571.1

30686-31027	113	Hp POR16_24233 (Ps pv. oryzae 1_6)	113/113(100%), 113/113(100%)	ZP_04590417.1
31101-32384	427	Phosphopyruvate hydratase (Ps pv. syringae B728a)	427/427(100%), 427/427(100%)	YP_234570.1
32500-33045	181	Inorganic diphosphatase (Ps pv. syringae B728a)	181/181(100%), 181/181(100%)	NP_794366.1
33045-33647	200	IS1415 transposase istA/B / Uracil DNA glycosylase superfamily protein (*Pseudomonas*)	199/200(99%), 200/200(100%)	WP_003317532.1
33656-34825	389	C4-dicarboxylate transporter/malic acid transport protein (Ps pv. syringae B728a)	389/389(100%), 389/389(100%)	YP_234567.1
34943-35839	298	LysR family transcriptional regulator (Ps pv. tomato DC3000)	298/298(100%), 298/298(100%)	NP_794363.1
35949-36989	346	Surface antigen (Ps pv. actinidiae)	346/346(100%), 346/346(100%)	gb|AGE82241.1
37319-37699	126	Hp Psyr_1476 (Ps pv. syringae B728a)	126/126(100%), 126/126(100%)	YP_234564.1
38414-39376	320	Phage integrase:Phage integrase, N-terminal SAM-like (Ps pv. syringae B728a)	320/320(100%), 320/320(100%)	YP_234563.1
39786-40112	108	Phage-related protein (*P. syringae*)	105/108(97%), 107/108(99%)	SFO59799.1
40224-40511	95	Hp (*Pseudomonas* sp. GM84), HTH domain,XRE superfamily	61/93(66%), 78/93(83%)	WP_008096879.1
40701-41474	257	LuxR family transcriptional regulator (Ps pv. tabaci ATCC 11528)	254/257(99%), 257/257(100%)	ZP_05639811.1
41565-42683	372	ABC transporter, periplasmic polyamine-binding protein (Ps pv. tomato NCPPB 1108)	364/372(98%), 370/372(99%)	ZP_07256281.1
42780-42977	65	Hp POR16_16968 (Ps pv. oryzae 1_6)	63/65(97%), 65/65(100%)	ZP_04588979.1
42989-43384	131	XRE/PbsX family transcriptional regulator (Ps BRIP39023)	127/131(97%), 129/131(98%)	ZP_20926098.1
43528-44112	194	Transcription termination factor nusG (*P. syringae*)	194/194(100%), 194/194(100%)	SDO17122.1
44201-44443	80	Hp PsyrptA_21031 (Ps pv. tabaci ATCC 11528)	78/80(98%), 79/80(98%)	ZP_05639806.1
44456-45163	235	VirB1 (Ps pv. aceris M302273)	218/235(93%), 224/235(95%)	ZP_16735663.1
45160-45498	112	VirB2 (Ps pv. oryzae 1_6)	110/111(99%), 110/111(99%)	ZP_04588984.1
45511-45987	158	VirB3 (VirB3) (*P. syringae*)	151/158(96%), 155/158(98%)	YP_006963339.1
45848-48400	850	VirB4 (Ps pv. phaseolicola 1448A)	820/850(96%), 833/850(98%)	YP_272283.1
48397-49083	228	VirB5 (Ps pv. syringae)	224/228(98%), 227/228(99%)	NP_940727.1
49112-49465	117	Hp POR16_17013 (Ps pv. oryzae 1_6)	101/117(86%), 108/117(92%)	ZP_04588988.1
49476-50423	315	VirB6 (Ps pv. oryzae 1_6)	289/315(92%), 302/315(95%)	ZP_04588989.1
50506-50808	100	VirB7 (*P. syringae* BRIP39023)	99/100(99%), 99/100(99%)	ZP_20926108.1
50846-51634	262	VirB8 (Ps pv. oryzae 1_6)	259/262(99%), 260/262(99%)	ZP_04590027.1
51624-52433	269	VirB9 (Ps pv. oryzae 1_6)	265/269(99%), 266/269(98%)	ZP_04590026.1
52420-53790	456	VirB10 (Ps pv. tabaci ATCC 11528)	436/456(96%), 442/456(96%)	ZP_05639796.1
53800-54831	343	VirB11 (Ps pv. syringae)	340/343(99%), 342/343(99%)	gb|AAR02182.1
54841-55197	118	Hp PsyrptN_02705 (Ps pv. tomato NCPPB 1108)	117/118(99%), 118/118(100%)	ZP_07256265.1
55323-57713	796	Hp PSPPH_B0039 (Ps pv. phaseolicola 1448A)	755/793(95%), 773/793(97%)	YP_272294.1
57800-58030	76	Hp (*P. syringae*)	69/76(91%), 73/76(96%)	WP_003407089.1
58030-59682	550	VirD4 (Ps pv. tomato NCPPB 1108)	509/550(93%), 529/550(96%)	ZP_07256262.1
59723-60085	120	Killer protein (Ps pv. aceris M302273PT)	119/120(99%), 119/120(99%)	ZP_16735648.1
60113-62278	721	Orf50 (Ps pv. syringae)	718/721(99%), 720/721(99%)	NP_940735.1
62307-62591	94	Hp Pav631_0804 (Pseudomonas avellanae BPIC 631)	88/94(94%), 89/94(94%)	ZP_16384506.1
62619-63221	200	Single-stranded DNA-binding protein (Ps pv. syringae)	199/201(99%), 199/201(99%)	NP_940736.1
63527-64261	244	Stability protein type I (Ps pv. tabaci ATCC 11528)	232/244(95%), 239/244(97%)	ZP_05639853.1
64258-64830	190	Hp PsyrptA_21261 (Ps pv. tabaci ATCC 11528)	183/190(96%), 187/190(98%)	ZP_05639852.1
64857-65279	140	Hp PMA4326A45 (Ps pv. maculicola)	109/139(78%), 120/139(86%)	YP_025663.1

65623-66348	241	Orf53 (*P. syringae*)	235/241(98%), 239/241(99%)	YP_006963255.1
66338-70096	1252	Orf54 (Ps pv. syringae)	1165/1256(93%), 1197/1256(95%)	NP_940739.1
70225-70875	216	ParA (Ps pv. oryzae 1_6)	216/216(100%), 216/216(100%)	ZP_04590005.1
70866-71219	117	Hp (*P. syringae*)	115/117(98%), 116/117(99%)	YP_006963258.1
71262-71426	54	Hp (*P. syringae*)	47/54(87%), 50/54(92%)	YP_006963259.1

[a] P: *Pseudomonas*, Ps: *Pseudomonas syringae*, Hp: Hypothetical protein. [b] Gray boxes: Specific genes present in this plasmid.

Table S8 Predicted ORFs in the *P. syringae* pv. garcae 2708 plasmid

Position (5´-3´)	Size (aa)	Homology (Blast)[a]	Identity and similarity (source of homolog)	Accession No.
1-1314	437	RepA (Ps pv. tomato)	422/437(97%), 428/437(97%)	emb|CAB37316.1
1585-1878	97	RulA (*P. savastanoi* pv. phaseolicola)	95/97(98%), 96/97(98%)	KPB47494.1
1930-2040	36	RulB (Ps pv. viburni)	26/34(76%), 28/34(82%)	gb|KPZ20060.1
2244-2831	195	Hp PsyrpaN_20016 (Ps pv. aesculi NCPPB 3681)	195/195(100%), 195/195(100%)	ZP_06460341.1
3273-4781[b]	502	Transposase (Ps pv. avellanae ISPaVe037)	501/502(99%), 502/502(100%)	ZP_17809899.1
4774-5577	267	ISPsy4, transposition helper protein (Ps pv. avellanae ISPaVe037)	267/267(100%), 267/267(100%)	ZP_17809900.1
5731-6216	161	Hp (Ps pv. maculicola)	157/160(98%), 159/160(99%)	YP_025681.1
6404-6676	90	Hp (Ps pv. maculicola)	90/90(100%), 90/90(100%)	YP_025682.1
6771-7418	215	Resolvase (*P. amygdali*)	207/215(96%), 211/215(98%)	ZP_11566872.1
7688-8344	218	ParA (Ps pv. glycinea B076)	216/218(99%), 218/218(100%)	ZP_11566906.1
8392-8646	84	Hp PsgB076_28900 (Ps pv. glycinea B076)	82/84(98%), 82/84(97%)	ZP_11566907.1
9039-9578	179	Hp PSYMP_27833 (Ps pv. morsprunorum M302280)	159/179(89%), 172/179(96%)	ZP_16661143.1
9838-10479	213	Hp PSPPH_B0011 (Ps pv. phaseolicola 1448A)	195/213(92%), 205/213(96%)	YP_272266.1
10854-11102	82	Hp PSYPI_16575 [Ps pv. pisi 1704B]	77/84(92%), 80/84(95%)	ZP_16696458.1
11470-11703	77	Hp PSYPI_29904 (Ps pv. pisi 1704B)	54/76(71%), 57/76(75%)	ZP_16698838.1
11716-11967	83	Hp A988_08589 (*P. syringae* BRIP39023)	82/83(99%), 83/83(100%)	ZP_20922757.1
11977-12705	242	Hp Patl_0675 (*Psal. atlantica* T6c)	99/243(41%), 143/243(58%)	YP_660257.1
12733-12921	62	Hp (*P. syringae*)	59/62(95%), 62/62(100%)	WP_003433293.1
12971-13267	98	Hp PLA107_32181 (Ps pv. lachrymans M301315)	50/98(51%), 62/98(63%)	ZP_16673571.1
13373-13516	47	Hp (*P. syringae*)	45/47(96%), 45/47(95%)	YP_006963299.1
13598-14500	300	HopBD1 (Ps pv. lachrymans M301315)	284/300(95%), 289/300(96%)	gb|ADQ74898.1
14930-15901	323	ExeA-like protein (Ps pv. maculicola)	322/323(99%), 323/323(100%)	YP_025679.1
15891-17549	552	Integrase (Ps pv. maculicola)	540/552(98%), 543/552(98%)	YP_025678.1
17533-18138	201	Resolvase (Ps pv. glycinea B076)	198/201(99%), 199/201(99%)	ZP_11566926.1
18678-18929	83	Hp A988_08589 (*P. syringae* BRIP39023)	80/83(96%), 82/83(98%)	ZP_20922757.1
18942-19349	135	Hp PMI25_01362 (*Pseudomonas* sp. GM30)	44/134(33%), 75/134(55%)	ZP_10679672.1
19868-20122	83	Antirestriction protein ArdR (*P. coronafaciens*)	83/83(100%), 83/83(100%)	KGS12384.1
21259-21900	213	Putative SOS response-associated peptidase YedK (*P. syringae*)	201/213(94%), 206/213(96%)	SDO16173.1
22021-22527	168	Hp PSYMP_28053 (Ps pv. morsprunorum M302280)	157/168(93%), 161/168(95%)	ZP_16661187.1
23204-23527	107	Stability/partitioning determinant (Ps pv. maculicola)	107/107(100%), 107/107(100%)	YP_025685.1
23520-24218	232	Plasmid partitioning protein ParA (*P. amygdali*)	231/232(99%), 231/232(99%)	WP_005734862.1
24639-24950	103	Plasmid stability protein StbC (Ps pv. tomato T1)	101/103(98%), 102/103(99%)	ZP_03400365.1
24947-25366	139	Plasmid stability protein StbB (Ps pv. tomato DC3000)	139/139(100%), 139/139(100%)	NP_808606.1
25407-26039	210	Resolvase (*P. syringae*)	207/210(99%), 207/210(98%)	gb|AAB81646.1
26314-27249	311	Syringolide biosynthetic protein AvrD1 (Ps pv. phaseolicola 1448A)	305/311(98%), 308/311(99%)	YP_272223.1
27489-28094	201	Phosphoglycerate mutase family protein (Ps pv. mori 301020)	192/196(98%), 193/196(98%)	ZP_16681154.1
29578-31773	731	Type III effector HopAU1 (Ps pv. theae)	725/731(99%), 728/731(99%)	gb|AEV42014.1
32325-32936	203	ExeA-like protein (*P. coronafaciens* pv. garcae)	200/203(99%), 201/203(99%)	KPX31711.1
33483-33743	86	Hp (*P. syringae* group)	86/86(100%), 86/86(100%)	WP_050586573.1
34011-34493	160	Hp PSPTO_4733 (Ps pv. tomato DC3000)	147/160(92%), 153/160(95%)	NP_794472.1

35095-37236	713	Type III effector HopD1 (Ps pv. tomato T1)	675/713(95%), 687/713(96%)	ZP_03400316.1
37517-38455	312	RulB protein (Ps pv. aceris M302273)	301/312(96%), 310/312(99%)	ZP_16735701.1
38780-39451	223	IS222 transposase orfB (Ps pv. maculicola)	209/221(95%), 217/221(98%)	YP_025628.1
39634-39942	102	ISPsy13, transposase OrfA (*P. avellanae* BPIC 631)	100/102(98%), 101/102(99%)	ZP_16384827.1
39982-40668	228	Type III effector AvrRps4 (*P. coronafaciens* pv. porri)	214/219(98%), 216/219(98%)	KOP53052.1
41188-41844	218	Type III effector HopH1 (*P. amydali* pv. mor)	215/218(99%), 217/218(99%)	KPY04699.1
42022-42246	74	Hp PsgB076_28905 (Ps pv. glycinea B076)	54/74(73%), 63/74(85%)	ZP_11566908.1
42269-43282	337	Phage integrase, N-terminal SAM-like protein (*P. syringae*)	306/337(91%), 317/337(94%)	YP_006963689.1
43449-43949	166	putative type VI secretion system effector, Hcp1 family (*P. amygdali* pv. lachrymans)	161/166(97%), 164/166(98%)	KPX58408.1
43974-44501	175	Hp PsgRace4_27800 (Ps pv. glycinea race 4)	159/175(91%), 165/175(94%)	ZP_11572227.1
44501-44788	95	Hp PsgRace4_27795 (Ps pv. glycinea race 4)	74/95(78%), 83/95(87%)	ZP_11572226.1
44816-45589	257	LuxR family transcriptional regulator (Ps pv. tabaci ATCC 11528)	218/257(85%), 240/257(93%)	ZP_05639811.1
45698-46798	366	ABC transporter, periplasmic polyamine-binding protein (Ps pv. tabaci ATCC 11528)	328/366(90%), 351/366(95%)	ZP_05639810.1
46895-47092	65	Hp PSYMP_24541 (Ps pv. morsprunorum M302280)	44/55(80%), 50/55(90%)	ZP_16666338.1
47110-47496	128	XRE/PbsX family transcriptional regulator (Ps pv. tomato Max13)	107/127(84%), 115/127(90%)	ZP_07234958.1
47712-48245	177	Transcriptional regulator/NusG protein	176/177(99%), 176/177(99%)	WP_024670607.1
48334-48576	80	Hp (Ps)	73/80(91%), 74/80(92%)	YP_006963336.1
48589-49323	244	VirB1 (Ps pv. pisi 1704B)	216/244(89%), 219/244(89%)	ZP_16698200.1
49320-49658	112	VirB2 (Ps pv. glycinea race 4)	111/112(99%), 112/112(100%)	ZP_16477757.1
49671-50159	162	VirB3 (Ps pv. syringae)	156/162(96%), 159/162(98%)	NP_940725.1
50008-52560	850	VirB4 (Ps pv. oryzae 1_6)	829/850(98%), 837/850(98%)	ZP_04588986.1
52557-53243	228	VirB5 (*P. syringae* BRIP39023)	208/228(91%), 218/228(95%)	ZP_20926105.1
53275-53628	117	Hp POR16_17013 (Ps pv. oryzae 1_6)	97/117(83%), 104/117(88%)	ZP_04588988.1
53639-54571	310	VirB6 (*P. syringae*)	277/310(89%), 293/310(94%)	YP_006963343.1
54638-54943	101	VirB7 (*P. syringae* BRIP39023)	93/103(90%), 96/103(93%)	ZP_20926108.1
54981-55769	262	VirB8 (*P. syringae*)	255/262(97%), 257/262(98%)	YP_006963345.1
55759-56568	269	VirB9 (*P. syringae*)	255/269(95%), 263/269(97%)	YP_006963346.1
56555-57913	452	VirB10 (Ps pv. glycinea B076)	396/452(88%), 420/452(92%)	ZP_11566994.1
57923-58981	352	VirB11 (*P. syringae*)	323/341(95%), 332/341(97%)	YP_006963348.1
58991-59347	118	Hp PMA4326A35 (Ps pv. maculicola)	114/118(97%), 118/118(100%)	YP_025653.1
59473-59715	80	Hp PSYAR_26419 (Ps pv. aceris M302273)	61/80(76%), 66/80(82%)	ZP_16735650.1
59715-61376	553	VirD4 (*P. syringae*)	525/553(95%), 541/553(97%)	YP_006963352.1
61416-61778	120	Killer protein (Ps pv. tabaci ATCC 11528)	110/120(92%), 113/120(94%)	ZP_05639857.1
61806-63971	721	Orf50 (Ps pv. tabaci ATCC 11528)	690/721(96%), 704/721(97%)	ZP_05639856.1
63998-64282	94	Hp Pav631_0804 (*P. avellanae* BPIC 631)	87/94(93%), 91/94(96%)	ZP_16384506.1
64310-64915	201	Single-strand DNA-binding protein (Ps pv. syringae)	187/201(93%), 193/201(96%)	NP_940736.1
64956-65240	94	Hp Psyrpa2_27382 (Ps pv. aesculi 2250)	72/94(77%), 78/94(82%)	ZP_06482774.1
65541-66275	244	Plasmid stability protein StbB(*P. coronafaciens*)	229/229(100%),229/229(100%)	KGS13871.1
66272-66844	190	Hp PSPSV_B0043 (*P. savastanoi*)	185/190(97%), 186/190(97%)	YP_006961620.1
66859-67248	129	Hp PsgB076_27670 (Ps pv. glycinea B076)	126/129(98%), 128/129(99%)	ZP_11566678.1
67603-67950	115	Hp I1A_000046 (*P. fluorescens* R124)	56/110(51%), 81/110(73%)	ZP_18349206.1

67934-71707	1257	Orf54 (*P. savastanoi*)	1070/1258(85%), 1154/1258(91%)	YP_006961623.1
71835-72263	142	GntR family transcriptional regulator (Ps pv. tomato DC3000)	133/142(94%), 136/142(95%)	NP_808662.1

[a] P: *Pseudomonas*, Ps: *Pseudomonas syringae*, Hp: Hypothetical protein. [b] Gray boxes: Specific genes present in this plasmid.

Table S9 Predicted ORFs in the *P. syringae* pv. tabaci 0893-29 plasmid

Position (5´-3´)	Size (aa)	Homology (Blast)[a]	Identity and similarity (source of homolog)	Accession No.
1-1314	437	RepA (Ps pv. tabaci)	436/437(99%), 437/437(100%)	gb|AAW01457.1
1453-1878	141	RulA (*P. savastanoi*)	140/141(99%), 141/141(100%)	gb|AAG39345.1
1856-3154	432	RulB (Ps pv. syringae)	394/432(91%), 4H0/432(94%)	NP_940693.1
3591-4064	157	Hp (*P. amygdali*)	156/157(99%), 157/157(100%)	WP_057413600.1
4843-5130	95	Hp ALO35_200076 (*P. amygdali* pv. lachrymans)	95/95(100%), 95/95(100%)	KPX70304.1
5204-5614	136	Hp (*P. amygdali*)	136/136(100%), 136/136(100%)	WP_057413602.1
5953-6153	66	Hp (*P. amygdali*)	66/66(100%), 66/66(100%)	WP_057413603.1
6264-6914	216	Chromosome partitioning prote ParA (Ps pv. maculicola M6)	212/216(98%), 216/216(100%)	NP_114201.1
6904-7191	95	Hp (*P. amygdali)*	95/95(100%), 95/95(100%)	WP_057413605.1
7429-8070	213	Hp ALO35_200173 (*P. amygdali* pv. lachrymans)	213/213(100%), 213/213(100%)	KPX70298.1
8168-9211	347	Hp (P. amygdali)	347/347(100%), 347/347(100%)	WP_057413607.1
10973-11557	194	Hp (*P. amygdali*)	194/194(100%), 194/194(100%)	WP_057413698.1
12479-12895	138	Hp (*P. amygdali*)	138/138(100%), 138/138(100%)	WP_057413696.1
13154-13444	96	Hp (*P. syringae* group)	96/96(100%), 96/96(100%)	WP_019331239.1
14053-14673	206	Resolvase type II (*P. savastanoi*)	206/206(100%), 206/206(100%)	WP_015060652.1
14984-15424	146	Hp Psyrpa2_22780 (Ps pv. aesculi 2250)	146/146(100%), 146/146(100%)	ZP_06481885.1
15858-16235	125	Hp PSPTOT1_3108 (Pspv. tomato T1)	125/125(100%), 125/125(100%)	ZP_03400175.1
16282-17118	278	Hp PsyrptK_27954 (Ps pv. tomato K40)	278/278(100%), 278/278(100%)	ZP_07255378.1
17176-17364	62	Hp (*P. amygdali*)	61/62(98%), 62/62(100%)	WP_057413695.1
17438-17716[b]	92	MobC (Ps pv. mori 301020)	91/92(99%), 92/92(100%)	ZP_16680680.1
17812-18015	67	Hp (*P. amygdali*)	67/67(100%), 67/67(100%)	WP_057413693.1
18162-18491	109	MobB (Ps pv. tomato DC3000)	109/109(100%), 109/109(100%)	NP_808686.1
18502-20457	651	MobA (Ps pv. aesculi NCPPB 3681)	636/651(98%), 643/651(98%)	ZP_06461231.1|
20500-20655	51	Integrase (*P. syringae*), Orf28	50/51(98%), 51/51(100%)	WP_017684530.1
20752-21594	280	LuxR family regulatory protein (Ps pv. aesculi 0893_23)	280/280(100%), 280/280(100%)	ZP_16660441.1
21652-22245	197	Anthranilate synthase component II (P spv. aesculi NCPPB 3681)	196/197(99%), 197/197(100%)	ZP_06461228.1
22242-23708	488	Anthranilate synthase component I (Ps pv. aesculi NCPPB 3681)	487/488(99%), 488/488(100%)	ZP_06461227.1
24337-25689	450	Phenylacetate-CoA ligase (*P. syringae* pv. actinidiae ICMP 18886)	450/450(100%), 450/450(100%)	EPM65131.1
25696-26241	181	GCN5-related N-acetyltransferase (Ps pv. aesculi NCPPB 3681)	180/181(99%), 180/181(99%)	ZP_06461226.1
26243-26926	227	TenA (Ps pv. aesculi NCPPB 3681)	227/227(100%), 227/227(100%)	ZP_06461225.1
26930-27547	205	Nadph-dependent fmn reductase (*P. syringae* pv. actinidiae ICMP 18886)	205/205(100%), 205/205(100%)	EPM65134.1
27550-28458	302	EamA permease drug metabolite transporter superfamily protein [*P. syringae* pv. actinidiae ICMP 18886]	302/302(100%), 302/302(100%)	EPM65135.1
28445-29395	316	Lipase (P. syringae pv. actinidiae ICMP 18801)	316/316(100%), 316/316(100%)	EPN74858.1
29811-30068	85	Hp (*P. syringae*)	83/85(98%), 85/85(100%)	WP_017704048.1
30365-30502	45	Hp (P. amygdali)	45/45(100%), 45/45(100%)	WP_005746560.1
30584-31486	300	HopBD1 (Ps pv. lachrymans M301315)	299/300(99%), 299/300(99%)	gb|ADQ74898.1
31921-32118	65	Hp PsyrptK_28690 (Ps pv. tomato K40)	60/65(92%), 63/65(96%)	ZP_07255519.1
32723-34018	431	Levansucrase LscC (Ps pv. glycinea race 4)	429/431(99%), 429/431(99%)	ZP_11567761.1
34111-34587	158	Hp PsyrpaN_18379 (Ps pv. aesculi NCPPB 3681)	157/158(99%), 157/158(99%)	ZP_06460034.1
34711-36660	649	GGDEF domain/EAL domain-containing protein (Ps pv. phaseolicola 1448A)	629/649(97%), 642/649(98%)	YP_272209.1

36711-36953	80	Hp PLA106_28151 (Ps pv. lachrymans M302278)	80/80(100%), 80/80(100%)	ZP_16718085.1
37276-37977	233	GntR family transcriptional regulator (Ps pv. tomato DC3000)	232/233(99%), 233/233(100%)	NP_808693.1
38046-39239	397	Hydroxyglutarate oxidase (Ps pv. morsprunorum M302280)	397/397(100%), 397/397(100%)	ZP_16666340.1
39280-40626	448	Major facilitator family transporter (Ps pv. tomato DC3000)	447/448(99%), 447/448(99%)	NP_808695.1
40667-40852	61	Hp PSYMP_24541 (Ps pv. morsprunorum M302280)	61/61(100%), 61/61(100%)	ZP_16666338.1
40874-41257	127	PbsX family transcriptional regulator (Ps pv. tomato Max13)	127/127(100%), 127/127(100%)	ZP_07234958.1
41472-42005	177	Transcriptional regulator (*P. amygdali*) NusG protein	177/177(100%) 177/177(100%)	WP_057413584.1
41995-42303	102	Hp PSPTO_B0047 (Ps pv. tomato DC3000)	100/102(98%), 101/102(99%)	NP_808633.1
42321-42593	90	Lipoprotein (Ps pv. tomato T1)	90/90(100%), 90/90(100%)	ZP_03400159.1
42596-43087	163	PilT protein (Ps pv. pisi 1704B)	163/163(100%), 163/163(100%)	ZP_16696502.1
43124-43597	157	TraH protein (Ps pv. tomato T1)	157/157(100%), 157/157(100%)	ZP_03399865.1
43825-44394	189	TraI (*P. avellanae* BPIC 631)	187/189(99%), 189/189(100%)	ZP_16388413.1
44391-45572	393	TraJ (Ps pv. glycinea B076)	388/393(99%), 390/393(99%)	ZP_11566802.1
45701-46354	217	Hp PSYPI_16815 (Ps pv. pisi 1704B)	211/215(98%), 212/215(98%)	ZP_16696506.1
46351-46902	183	Endonuclease (Ps pv. tomato T1)	181/183(99%), 182/183(99%)	ZP_03399861.1
46939-47184	81	Hp PsgRace4_27620 (Ps pv. glycinea race 4)	81/81(100%), 81/81(100%)	ZP_11572192.1
47213-47500	95	TraK protein (Ps pv. tomato DC3000)	95/95(100%), 95/95(100%)	NP_808642.1
47543-52174	1543	DNA primase (Ps pv. glycinea B076)	1522/1543(99%), 1528/1543(99%)	ZP_11566797.1
52200-52553	117	TraL protein (Ps pv. morsprunorum M302280)	115/117(98%), 115/117(98%)	ZP_16666322.1
52472-53257	261	TraM protein (Ps pv. tomato T1)	259/261(99%), 260/261(99%)	ZP_03399857.1
53314-54372	352	TraN protein (Ps pv. tomato T1)	350/352(99%), 351/352(99%)	ZP_03399856.1
54378-55736	452	TraO protein (Ps pv. glycinea B076)	449/451(99%), 449/451(99%)	ZP_11566793.1
55733-56446	237	TraP protein (Ps pv. tomato DC3000)	227/237(96%), 230/237(97%)	NP_808648.1
56455-56997	180	TraQ protein (Ps pv. tomato DC3000)	180/180(100%), 180/180(100%)	NP_808713.1
57073-57468	131	TraR protein (Ps pv. tomato Max13)	131/131(100%), 131/131(100%)	ZP_07234267.1
57549-58160	203	TraT protein (Ps pv. pisi 1704B)	201/203(99%), 201/203(99%)	ZP_16696516.1
58135-61191	1018	TraU protein (Ps pv. glycinea B076)	1008/1018(99%), 1015/1018(99%)	ZP_11566786.1
61202-62416	404	TraW protein (Ps pv. tomato DC3000)	402/404(99%), 404/404(100%)	NP_808652.1
62406-63008	200	TraX protein (Ps pv. lachrymans M302278)	197/200(99%), 197/200(98%)	ZP_16722489.1
63039-65183	714	TraY protein (Ps pv. pisi 1704B)	700/714(98%), 706/714(98%)	ZP_16696520.1
65285-65530	81	Hp PsgB076_28220 (Ps pv. glycinea B076)	80/81(99%), 80/81(98%)	ZP_11566781.1
65526-66176	216	Surface exclusion protein, putative (Ps pv. glycinea race 4)	204/216(94%), 210/216(97%)	ZP_11572174.1
66270-67472	400	TrbA protein (*P. avellanae* BPIC 631)	397/400(99%), 399/400(99%)	ZP_16388390.1
67462-67734	90	PilT domain-containing protein (*P. avellanae* BPIC 631)	90/90(100%), 90/90(100%)	ZP_16388391.1
67836-68036	66	Hp PSPPH_A0035 (Ps pv. phaseolicola 1448A)	66/66(100%), 66/66(100%)	YP_272158.1
68128-69213	361	TrbB protein (Ps pv. glycinea race 4)	354/361(98%), 357/361(98%)	ZP_11572170.1
69200-71488	762	TrbC protein (Ps pv. pisi 1704B)	757/762(99%), 758/762(99%)	ZP_16696527.1
71516-71938	140	Hp PSPTOT1_4425 (Ps pv. tomato T1)	139/140(99%), 139/140(99%)	ZP_03399839.1
71959-72522	187	Hp Pav631_5140 (*P. avellanae* BPIC 631)	184/187(98%), 185/187(98%)	ZP_16388449.1
72710-73138	142	GntR family transcriptional regulator (Ps pv. tomato T1)	142/142(100%), 142/142(100%)	ZP_03399837.1
73217-73444	75	Hp PsyrpaN_22863 (Ps pv. aesculi NCPPB 3681)	75/75(100%), 75/75(100%)	ZP_06460896.1

[a] P: *Pseudomonas*, Ps: *Pseudomonas syringae*, Hp: Hypothetical protein. [b] Gray boxes: Specific genes present in this plasmid.
